# Supplementary material for: On convolutional neural networks for selection inference: Revealing the effect of preprocessing on model learning and the capacity to discover novel patterns
Source: PLoS Comput Biol. 2023 Nov 27;19(11):e1010979. doi: 10.1371/journal.pcbi.1010979 (PMC10703409; doi:10.1371/journal.pcbi.1010979)
Supplement: S1 Text — Fig A. Comparison of Imagene with mini-CNN. Table A. Model accuracy for decreasing levels of complexity, for single-population demographic model with selection coefficient s = 0.01. Fig B. SHAP explanations for Imagene predictions without row-sorting. Table B. Model accuracy for decreasing levels of complexity, for single-population demographic model with selection coefficient s = 0.01, trained without row-sorting. Fig C. Performance correlation for summary statistics and CNN approaches under image resizing. Fig D. Visualization of model performances across all demographic models for selection coefficient of 0.01. Fig E. Visualization of model performances across all demographic models for selection coefficient of 0.005. Fig F. Simulations of CEU and YRI under the three-population demographic model match the site frequency spectrum of 1000 Genomes populations. Fig G. SHAP explanations for Imagene predictions under image resizing. Fig H. Visualization of mini-CNN dense layer under image resizing. Fig I. Visualization of mini-CNN dense layer under zero-padding. Fig J. SHAP explanations for Imagene predictions under zero-padding. Fig K. Performance correlation for summary statistics and CNN methods under zero-padding. Fig L. SHAP explanations for Imagene predictions with trimming for standardizing image width. Fig M. Full images corresponding to the cropped images in Fig 4. Fig N. SHAP visualizations for Imagene trained to distinguish between hard/soft sweeps and neutrality. Fig O. Visualizations of mini-CNN dense layer, comparing Hard vs Neutral and Hard vs Soft sweep classification. Fig P. Sample Complexity Analysis. Fig Q. Visualization of model performances across all demographic models and training strategies, for selection coefficient of 0.01 Fig R. Visualization of model performances across all demographic models and training strategies, for selection coefficient of 0.005. (PDF) [file pcbi.1010979.s002.pdf]

Supplementary Material

On convolutional neural networks for selection inference: revealing the effect of preprocessing on model learning and the capacity to discover novel patterns

Ryan M. Cecil and Lauren A. Sugden

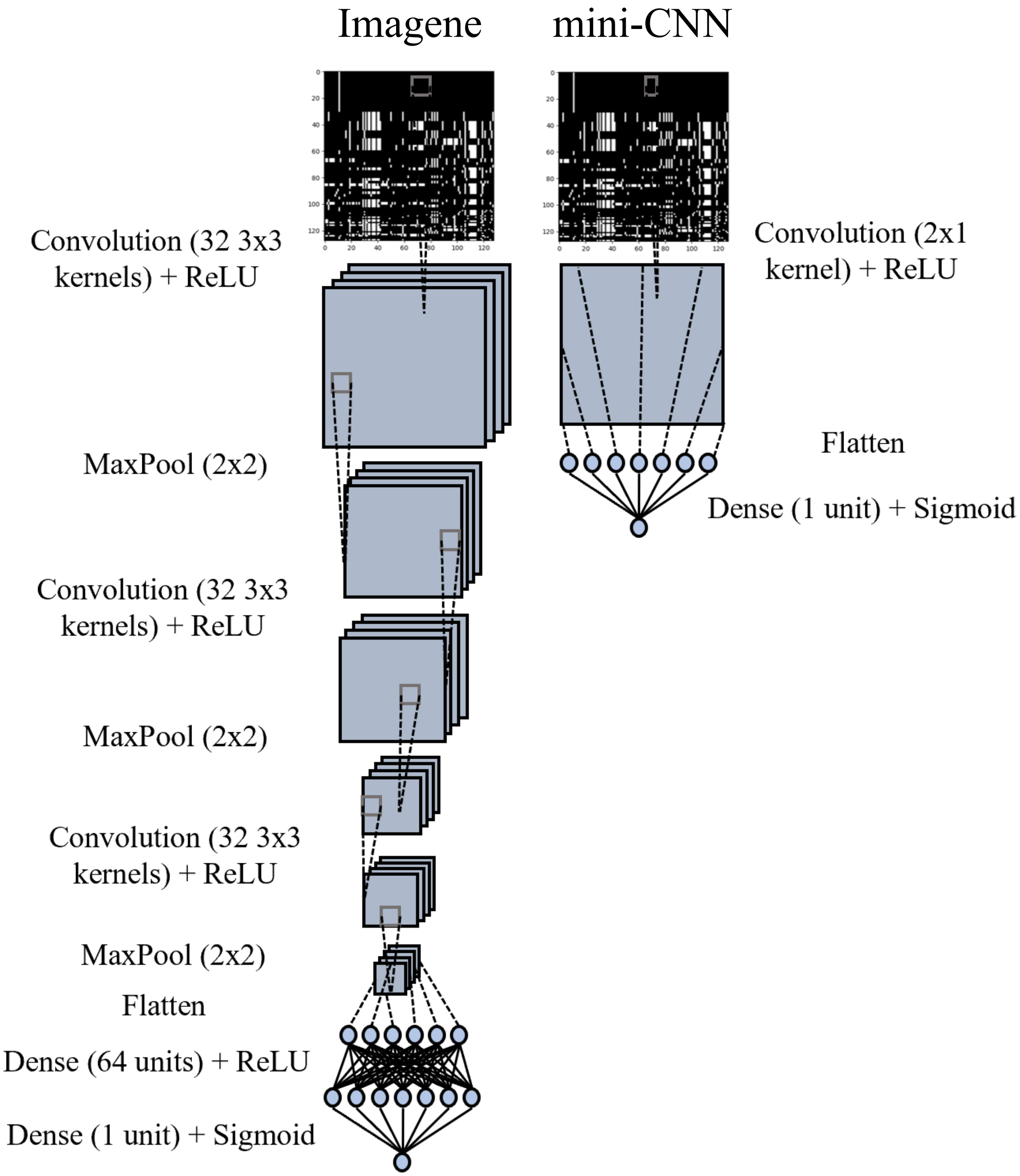

Figure A Comparison of Imagene with mini-CNN. Imagene, similar to other CNNs for detecting selection, contains multiple convolution layers and kernels. mini-CNN contains a single convolution layer with a single 2x1 kernel, followed by ReLU activation and a single dense layer.

	Architecture	Accuracy
**Imagene**	3 × [Conv2d(32 3x3 kernels) + ReLU + MaxPool(2x2)]<br>[Dense Layer (64 units) + ReLU]<br>[Dense Layer (1 unit) + Sigmoid]	97.6
	3 × [Conv2d(32 3x3 kernels) + ReLU + MaxPool(2x2)]<br>[Dense Layer (1 unit) + Sigmoid]	97.5
	2 × [Conv2d(32 3x3 kernels) + ReLU + MaxPool(2x2)]<br>[Dense Layer (1 unit) + Sigmoid]	98.0
	1 × [Conv2d(32 3x3 kernels) + ReLU + MaxPool(2x2)]<br>[Dense Layer (1 unit) + Sigmoid]	97.8
	1 × [Conv2d(16 3x3 kernels) + Relu + MaxPool(2x2)]<br>[Dense Layer (1 unit) + Sigmoid]	97.8
	1 × [Conv2d(4 3x3 kernels) + Relu + MaxPool(2x2)]<br>[Dense Layer (1 unit) + Sigmoid]	97.8
	1 × [Conv2d(1 3x3 kernel) + Relu + MaxPool(2x2)]<br>[Dense Layer (1 unit) + Sigmoid]	97.7
	1 × [Conv2d(1 2x2 kernel) + Relu + MaxPool(2x2)]<br>[Dense Layer (1 unit) + Sigmoid]	97.8
	1 × [Conv2d(1 1x2 kernel) + Relu + MaxPool(2x2)]<br>[Dense Layer (1 unit) + Sigmoid]	84.3
	1 × [Conv2d(1 2x1 kernel) + Relu + MaxPool(2x2)]<br>[Dense Layer (1 unit) + Sigmoid]	97.4
**mini-CNN**	1 × [Conv2d(1 2x1 kernel) + Relu]<br>[Dense Layer (1 unit) + Sigmoid]	97.3
	1 × [Conv2d(1 2x1 kernel) + MaxPool(2x2)]<br>[Dense Layer (1 unit) + Sigmoid]	97.3
	1 × [Conv2d(1 2x1 kernel)]<br>[Dense Layer (1 unit) + Sigmoid]	78.9
	[Dense Layer (1 unit) + Sigmoid]	77.6

Table A Model accuracy for decreasing levels of complexity, for single-population demographic model with selection coefficient $s = 0.01$. Each model is trained on 80,000 training simulations, with 20,000 simulations for testing and validation. Accuracy is calculated on a balanced testing set, and red values indicate substantial losses in accuracy. mini-CNN is one of two models with the lowest complexity that maintains high performance: 1 convolutional layer with a single 2x1 kernel, followed by either ReLu or MaxPooling, with a single 1-unit dense layer followed by a sigmoid function. We chose the model with ReLu rather than MaxPooling for its slight favorability with respect to interpretation of the model. We note that a 1x2 kernel does not perform as well (with 84.3% accuracy), indicating that row-to-row differences are a more salient signal than column-to-column differences, as would be expected. In addition, a single dense layer followed by a sigmoid, replicating a logistic regression model on the pixels of the input image, also does not perform as well (77.6% accuracy).

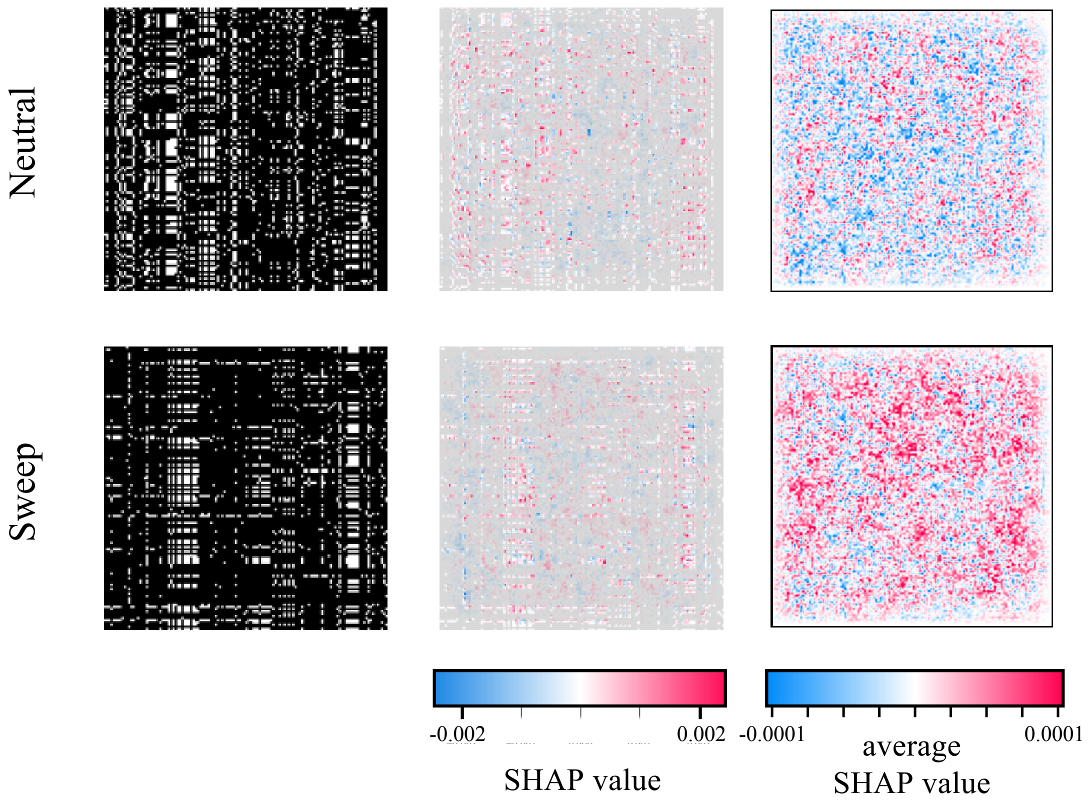

Figure B SHAP explanations for Imagene predictions without row-sorting. Visualization of Imagene with SHAP explanations. From left to right are examples of neutral and sweep processed images, SHAP values for the two image examples, and average SHAP values across 1000 neutral and sweep images. A negative SHAP value (blue) indicates that the pixel of interest contributes toward a prediction of neutral, while a positive SHAP value (red) indicates that the pixel of interest contributes toward a prediction of sweep. Without row-sorting, it is difficult to identify any particular patterns of interest to the model.

	Architecture	Accuracy
**Imagene**	3 × [Conv2d(32 3x3 kernels) + ReLU + MaxPool(2x2)]<br>[Dense Layer (64 units) + ReLU]<br>[Dense Layer (1 unit) + Sigmoid]	74.9
	3 × [Conv2d(32 3x3 kernels) + ReLU + MaxPool(2x2)]<br>[Dense Layer (1 unit) + Sigmoid]	71.9
	2 × [Conv2d(32 3x3 kernels) + ReLU + MaxPool(2x2)]<br>[Dense Layer (1 unit) + Sigmoid]	73.2
	1 × [Conv2d(32 3x3 kernels) + ReLU + MaxPool(2x2)]<br>[Dense Layer (1 unit) + Sigmoid]	71.6
	1 × [Conv2d(16 3x3 kernels) + Relu + MaxPool(2x2)]<br>[Dense Layer (1 unit) + Sigmoid]	72.2
	1 × [Conv2d(4 3x3 kernels) + Relu + MaxPool(2x2)]<br>[Dense Layer (1 unit) + Sigmoid]	73.4
	1 × [Conv2d(4 3x1 kernels) + Relu + MaxPool(2x2)]<br>[Dense Layer (1 unit) + Sigmoid]	54.7
	1 × [Conv2d(1 3x3 kernel) + Relu + MaxPool(2x2)]<br>[Dense Layer (1 unit) + Sigmoid]	52.5
	1 × [Conv2d(1 2x2 kernel) + Relu + MaxPool(2x2)]<br>[Dense Layer (1 unit) + Sigmoid]	53.4
	1 × [Conv2d(1 1x2 kernel) + Relu + MaxPool(2x2)]<br>[Dense Layer (1 unit) + Sigmoid]	53.1
	1 × [Conv2d(1 2x1 kernel) + Relu + MaxPool(2x2)]<br>[Dense Layer (1 unit) + Sigmoid]	53.2
**mini-CNN**	1 × [Conv2d(1 2x1 kernel) + Relu]<br>[Dense Layer (1 unit) + Sigmoid]	51.3
	1 × [Conv2d(1 2x1 kernel) + MaxPool(2x2)]<br>[Dense Layer (1 unit) + Sigmoid]	53.3
	1 × [Conv2d(1 2x1 kernel)]<br>[Dense Layer (1 unit) + Sigmoid]	51.5
	[Dense Layer (1 unit) + Sigmoid]	51.5

Table B Model accuracy for decreasing levels of complexity, for single-population demographic model with selection coefficient $s = 0.01$, trained without row-sorting. Each model is trained on 80,000 training simulations, with 20,000 simulations for testing and validation. Accuracy is calculated on a balanced testing set, and red values indicate substantial losses in accuracy. In the absence of row sorting, four 3x3 kernels appear to be necessary to maintain performance.

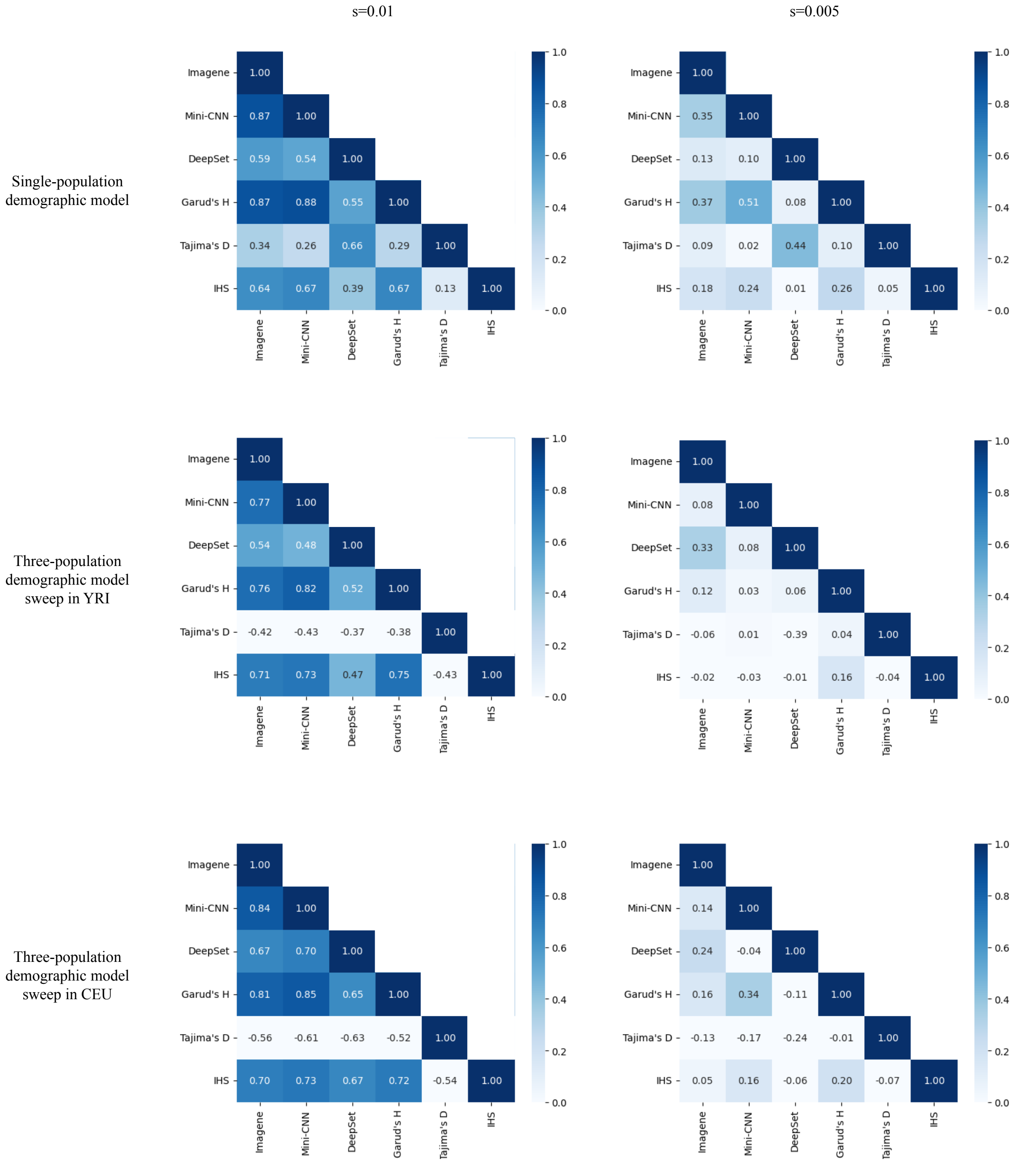

Figure C Performance correlation for summary statistics and CNN approaches under image resizing. Spearman correlation matrices are shown for the single population demographic model, as well as the three-population demographic model with sweeps in YRI and in CEU. Left matrices are calculated for sweep simulations with selection coefficient s=0.01, and right matrices are calculated for selection coefficient s=0.005. CNN methods (Imagene, mini-CNN, and DeepSet) are run on pre-processed images using image resizing.

	Single pop model		Three-pop model, sweep in YRI		Three-pop model, sweep in CEU		Three-pop model, sweep in CHB	
Selection coefficient (s)	0.01	0.005	0.01	0.005	0.01	0.005	0.01	0.005
Imagene	97.60	56.00	82.30	55.60	84.80	53.00	81.90	53.60
Mini-CNN	97.30	58.10	80.50	51.40	84.30	55.30	84.90	52.80
DeepSet	75.67	50.96	74.40	61.35	75.25	57.35	77.90	55.90
Imagene (ZP)	**98.20**	55.10	85.60	68.00	88.80	69.10	81.90	62.10
Mini-CNN (ZP)	97.60	58.20	**86.10**	73.20	87.20	68.50	84.90	66.30
DeepSet (ZP)	86.77	54.20	82.95	**75.70**	81.45	69.10	81.60	68.20
Imagene (tr)	92.55	55.87	79.00	54.05	50.00	59.20	77.30	57.70
Mini-CNN (tr)	92.03	55.00	74.75	53.1	77.7	61.05	78.6	60.05
DeepSet (tr)	66.00	52.65	65.30	59.85	75.25	56.55	76.55	55.80
Garud's H1	98.15	**61.07**	84.70	54.25	**89.15**	55.65	**88.25**	57.10
IHS	79.79	55.18	81.70	50.95	80.25	50.90	79.35	50.90
Tajima's D	64.80	52.08	50.00	50.00	50.00	50.00	50.00	50.10
S	81.90	53.36	81.15	74.65	81.85	**72.20**	78.95	**72.35**

Table C Model accuracy for all CNN models and summary statistics, across all demographic models and types of pre-processing. Performance values of multiple methods that were trained and tested on the same demographic model and selection coefficient. The performance values were found by computing the accuracy of the trained model or statistic on a balanced, held-out set. Here, (ZP) denotes that the images were standardized to a fixed width using zero padding, and (tr) indicates that the images were standardized to a fixed width using trimming. In all other cases where standardization was required, an image resizing algorithm was used.

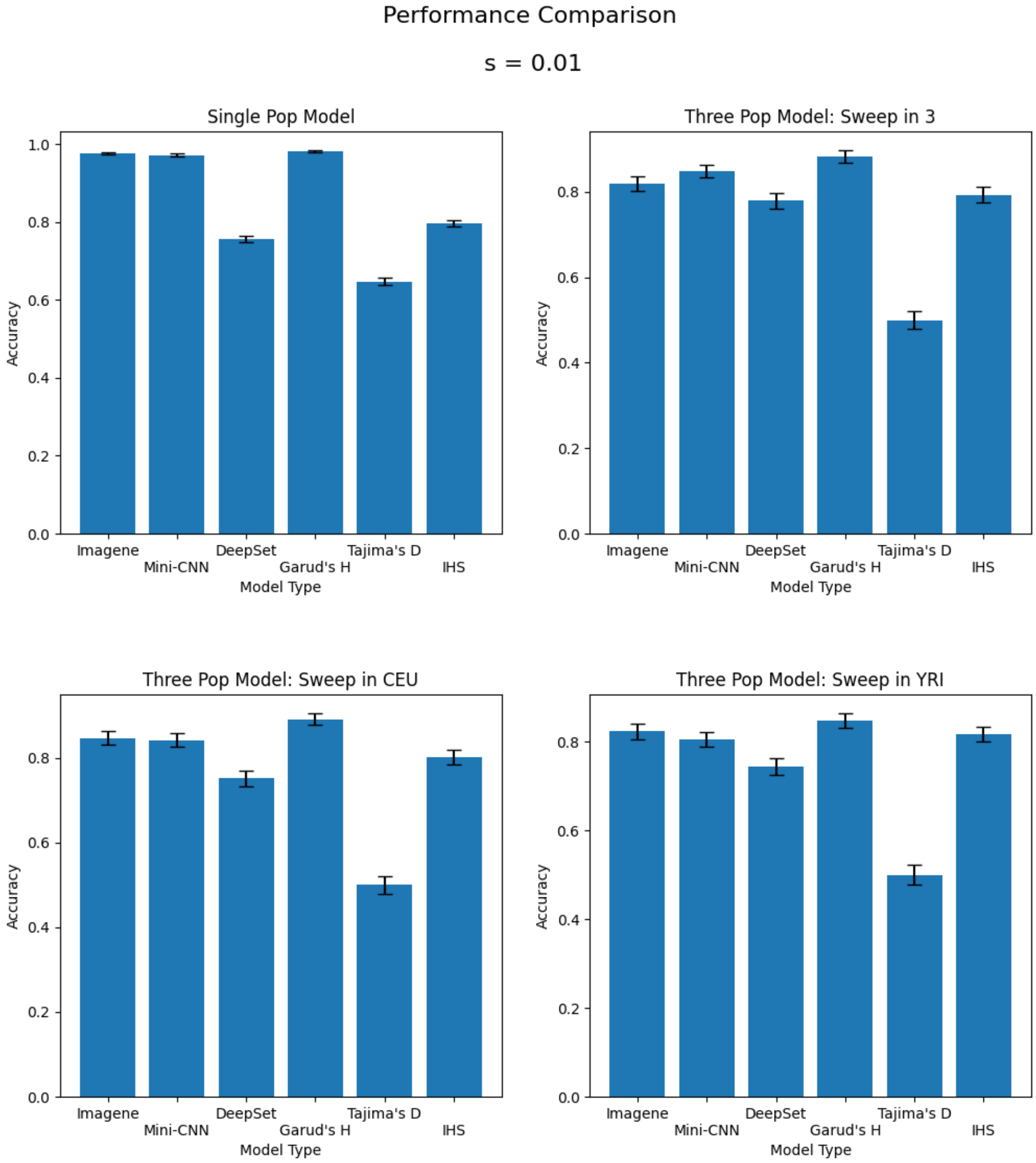

Figure D Visualization of model performances across all demographic models for selection coefficient of 0.01. Each sub-plot corresponds to a different demographic model, the x-axis denotes the model type, and the y-axis corresponds to the accuracy of the model on a balanced, held-out set. In all cases where image standardization was required, an image resizing algorithm was used. Error bars correspond to 95% confidence intervals for the accuracy on the test set.

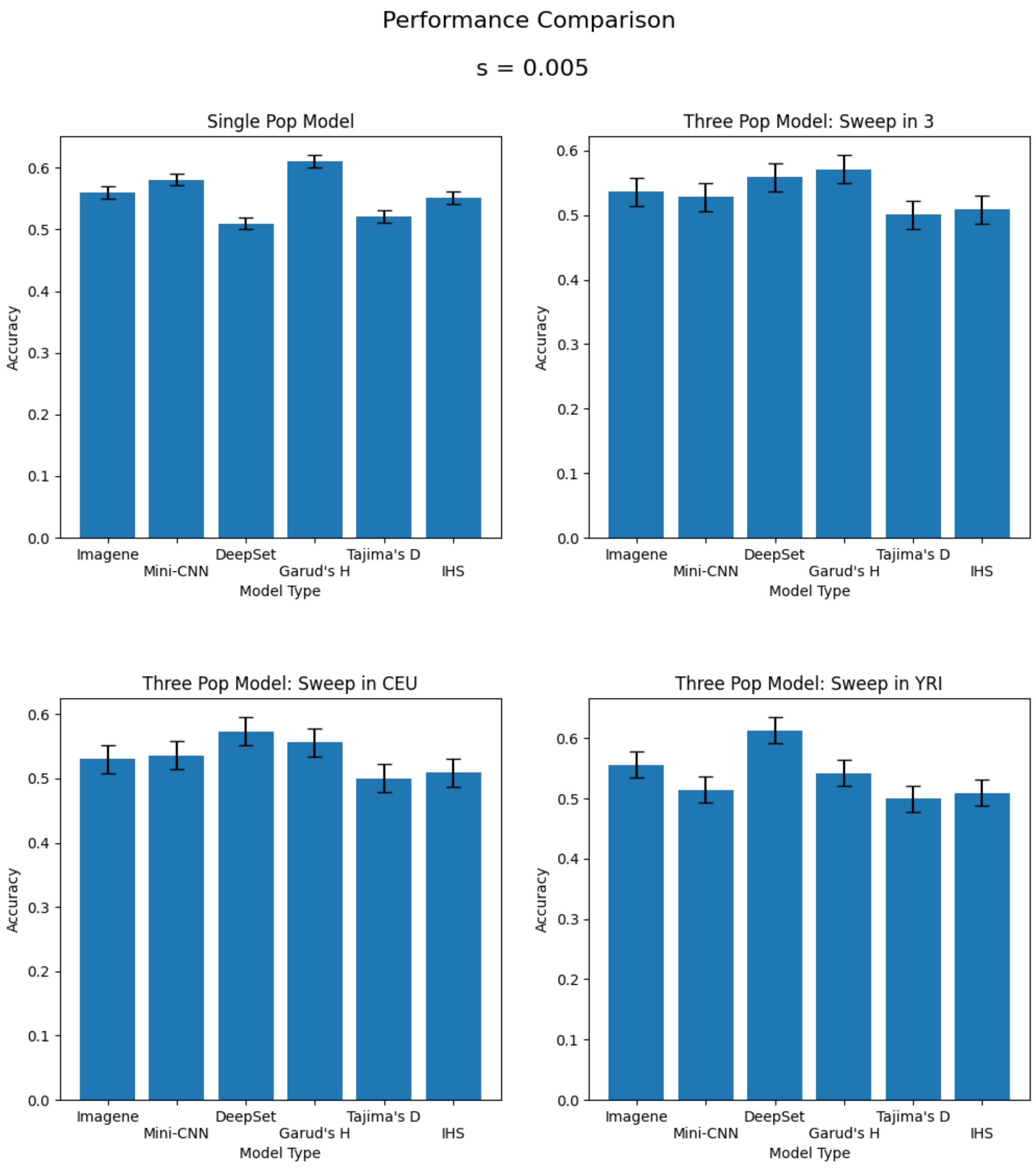

Figure E Visualization of model performances across all demographic models for selection coefficient of 0.005. Each sub-plot corresponds to a different demographic model, the x-axis denotes the model type, and the y-axis corresponds to the accuracy of the model on a balanced, held-out set. In all cases where image standardization was required, an image resizing algorithm was used. Error bars correspond to 95% confidence intervals for the accuracy on the test set.

Selection coefficient (s)	0.01		0.005	
Number of Haplotypes	128	1000	128	1000
Imagene	97.60	99.18	56.00	60.08
Mini-CNN	97.30	99.05	58.10	61.62
DeepSet	75.67	85.2	50.96	53.17
Garud's H1	**98.15**	**99.68**	**61.07**	**67.59**

Table D Model accuracy for Imagene, Mini-CNN, DeepSet, and Garud's H1 methods on Single Pop Model with varying selection coefficient and number of haplotypes. The performance values were found by computing the accuracy of the trained model or statistic on a balanced, held-out set of size 10,000. For image standardization with the ML models, an image resizing algorithm was used. The images with 128 haplotypes were resized to a height and width of 128x128. Due to GPU memory constraints, the images with 1000 haplotypes were resized to 200x200. The top performing method is bolded in each column.

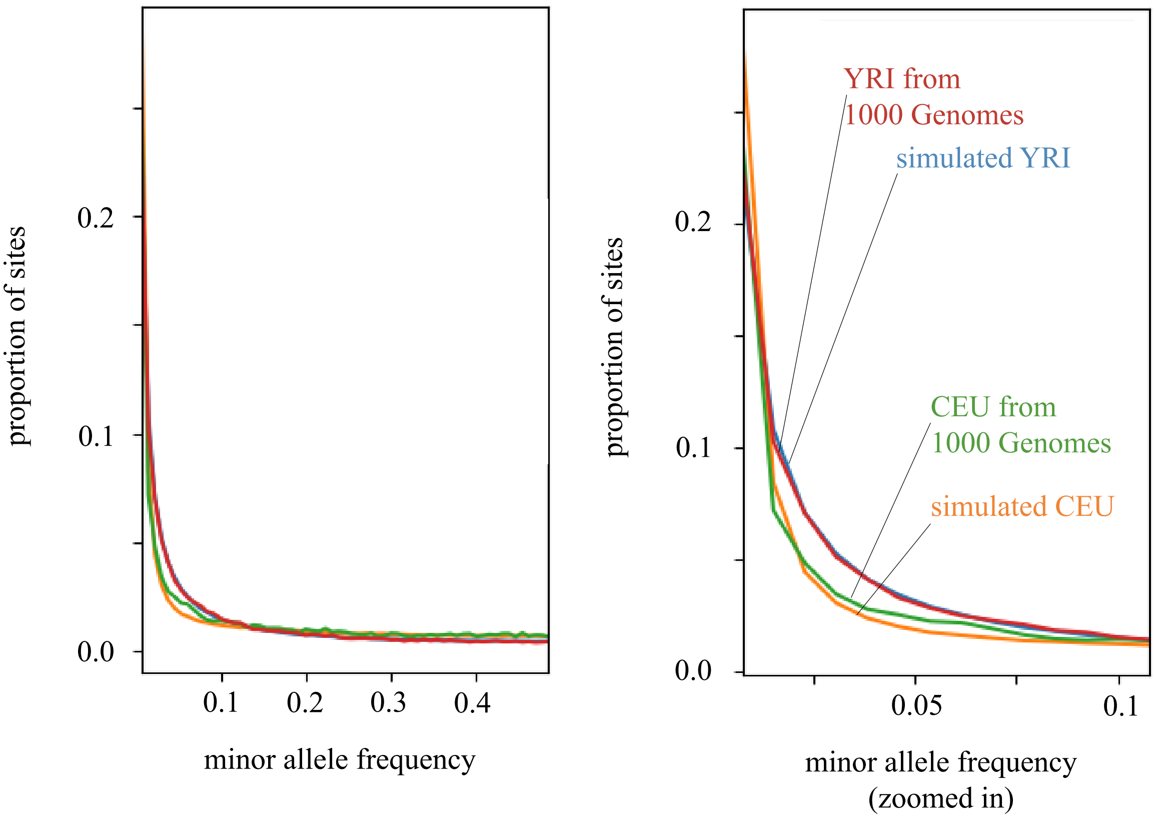

Figure F Simulations of CEU and YRI under the three-population demographic model match the site frequency spectrum of 1000 Genomes populations. On the left is the full folded site frequency spectrum(SFS) for each simulated dataset and real dataset, and on the right is the same figure, zoomed into the low minor allele frequencies for easier visualization. The SFS curves for the simulated populations match quite closely the observed SFS curves for each of the two populations, an indication that these simulations do a decent job of capturing the overall sequence diversity of these populations.

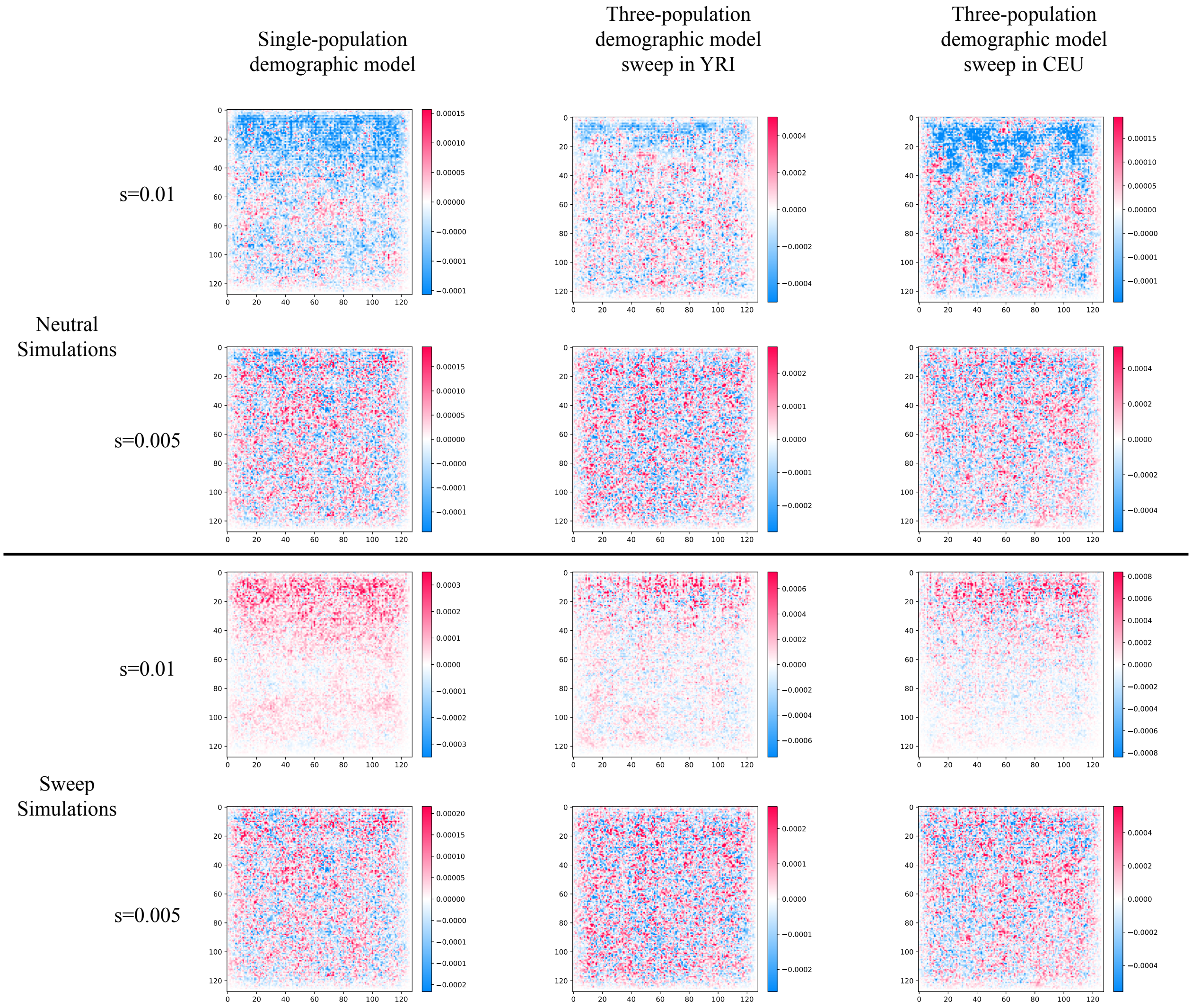

Figure G SHAP explanations for Imagene predictions under image resizing. SHAP explanations are shown for neutral and sweep simulations, across demographic models and selection coefficients. Dark red indicates pixels that are influential for classifying the image as a sweep, and dark blue indicates pixels that are influential for classifying the image as neutral. In general, the rows at the top of the image are informative for moderate selection coefficient $s = 0.01$ (1st and 3rd rows), but Imagene, like other methods, has trouble distinguishing between neutral and sweep simulations when the selection coefficient is low ($s = 0.005$; second and fourth rows).

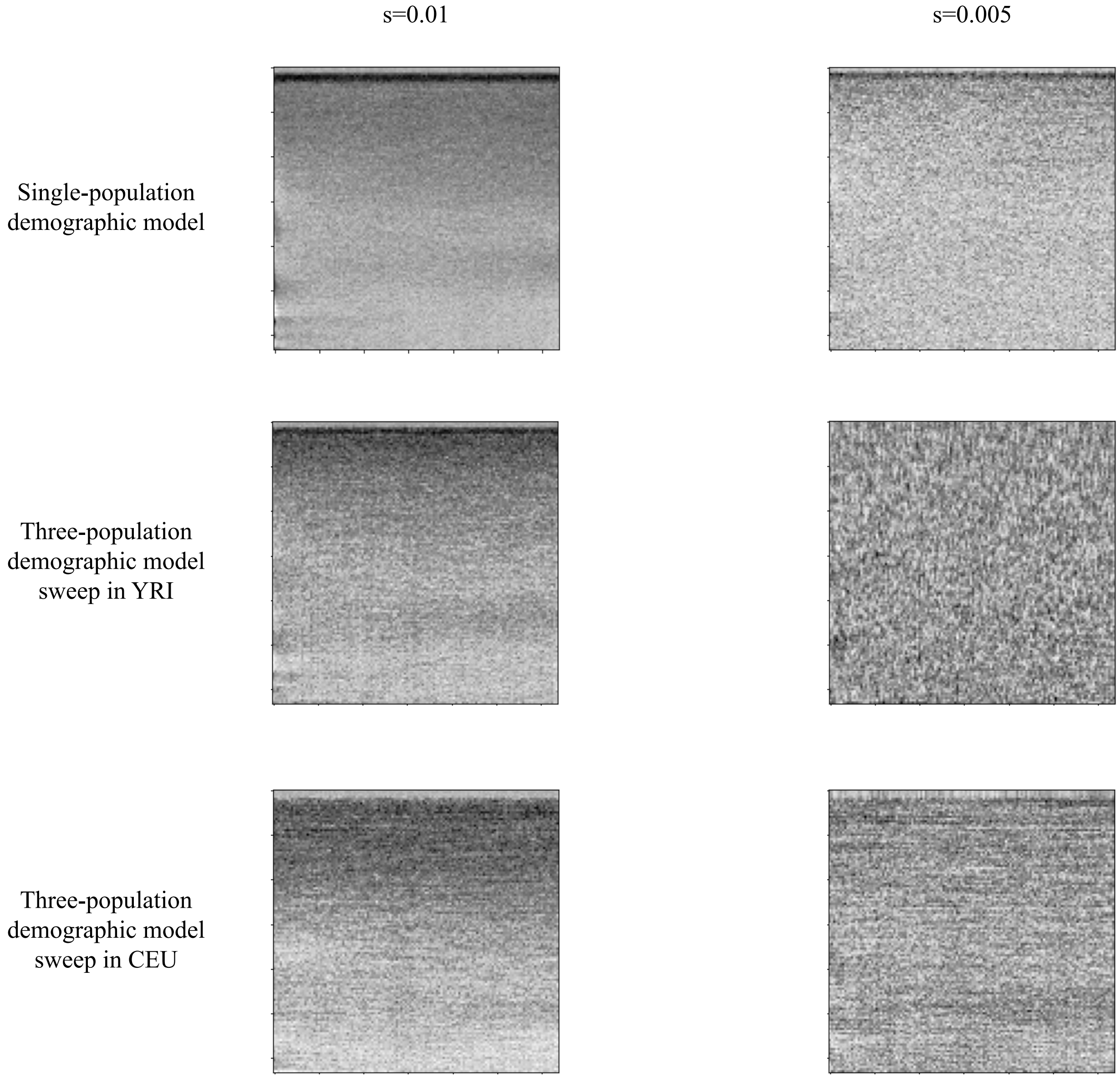

Figure H Visualization of mini-CNN dense layer under image resizing. mini-CNN shows similar patterns to those seen with Imagene in Figure G; for moderate selection coefficient $s = 0.01$, the most influential pixels are near the top of the image. For $s = 0.005$, mini-CNN has trouble discerning a signature in all but the simplest case of the single-population demographic model.

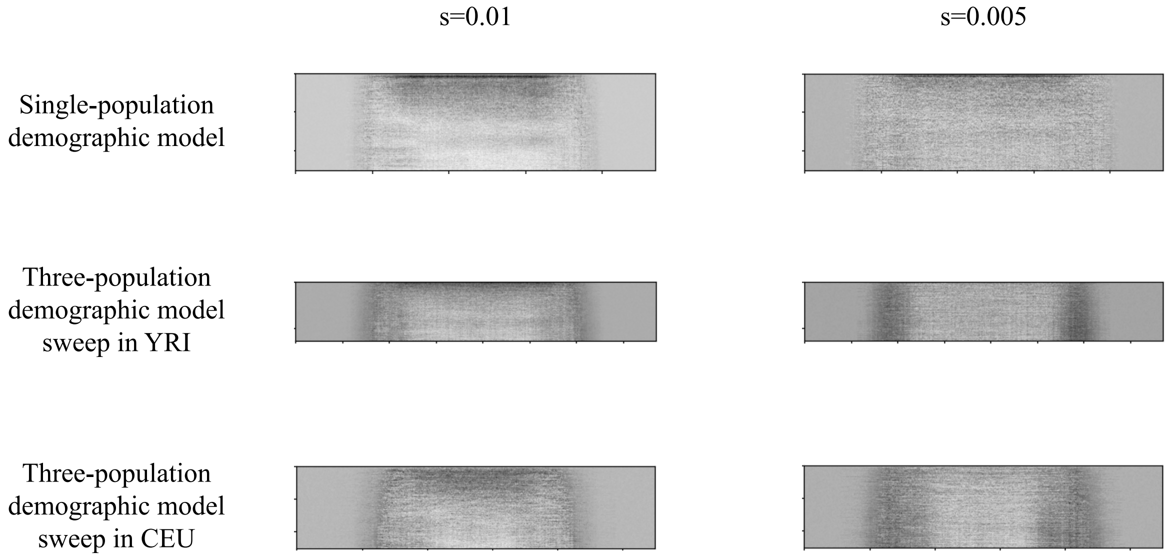

Figure I Visualization of mini-CNN dense layer under zero-padding. When zero-padding is used in preprocessing, the pixels that most influence classification tend to lie both at the top of the image, and in vertical strips, similar to the patterns seen in Figure J. Also similarly, this pattern is especially apparent when the selection coefficient is low.

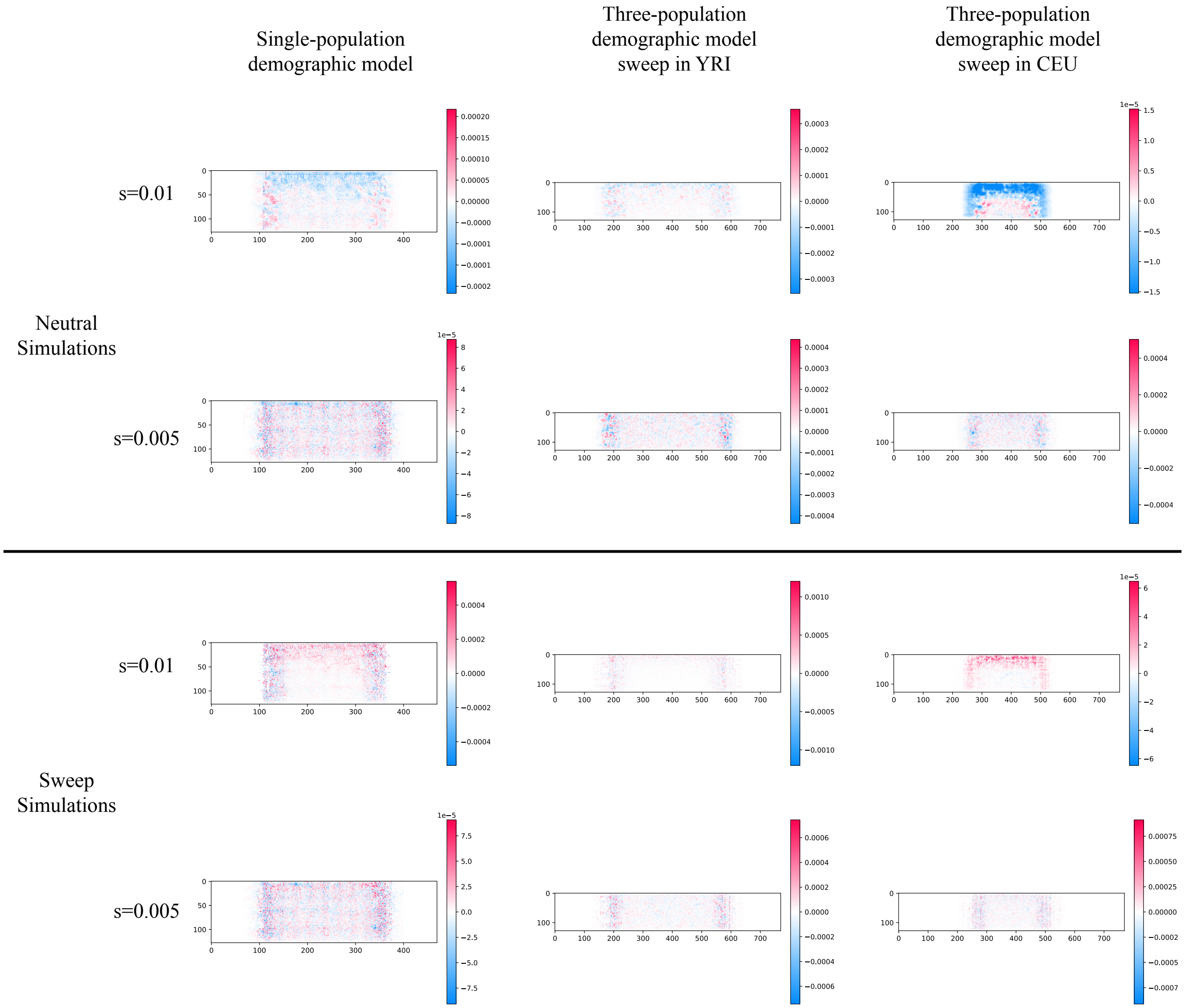

Figure J SHAP explanations for Imagene predictions under zero-padding. SHAP explanations are shown across demographic models and selection coefficients. Informative pixels tend to be at the top of the image as in Figure G, but also in vertical strips. Since neutral images tend to have a greater number of segregating sites, especially in simulated data that does not account for mutation rate variation, sweep images typically have more padded columns added. The CNN therefore learns that finding variation beyond a certain point is a signature of neutrality. We note that when the selection coefficient is low ($s = 0.005$; second and fourth rows), this paves the way for higher classification accuracy when compared with the accuracy on the same scenario under image resizing. We urge caution in generalizing this result, however, without analyzing performance on a wider range of simulations across a range of mutation rates.

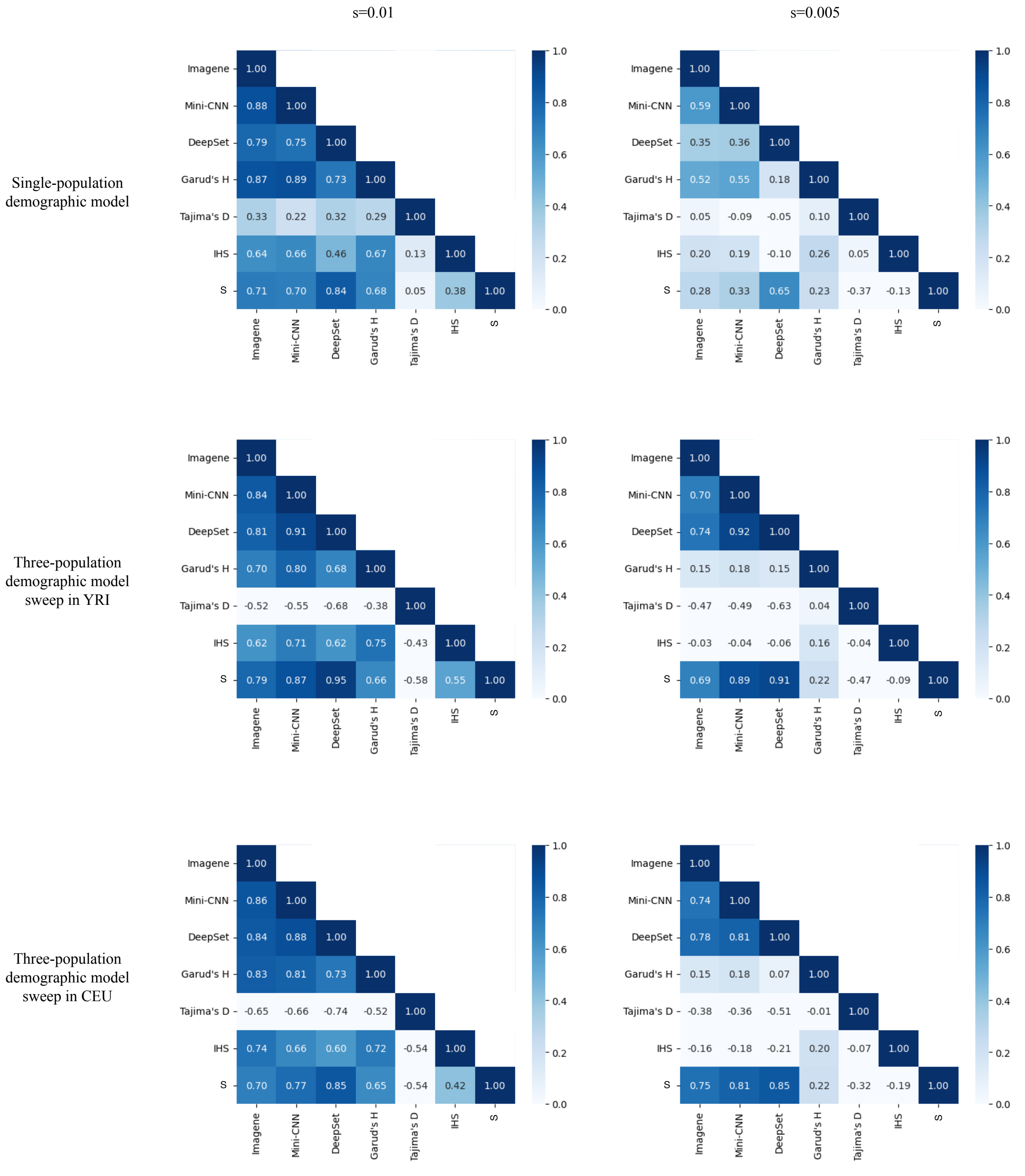

Figure K Performance correlation for summary statistics and CNN methods under zero-padding. Spearman correlation matrices are shown for the same models as in Figure C. CNN methods are run on pre-processed images using zero-padding. In addition, we include a summary statistic "Ncols" that counts the number of columns in the image after removal of sites with minor allele frequency below 1%; the correlation of Ncols with the CNN approaches illustrates the potential artifacts introduced by zero-padding.

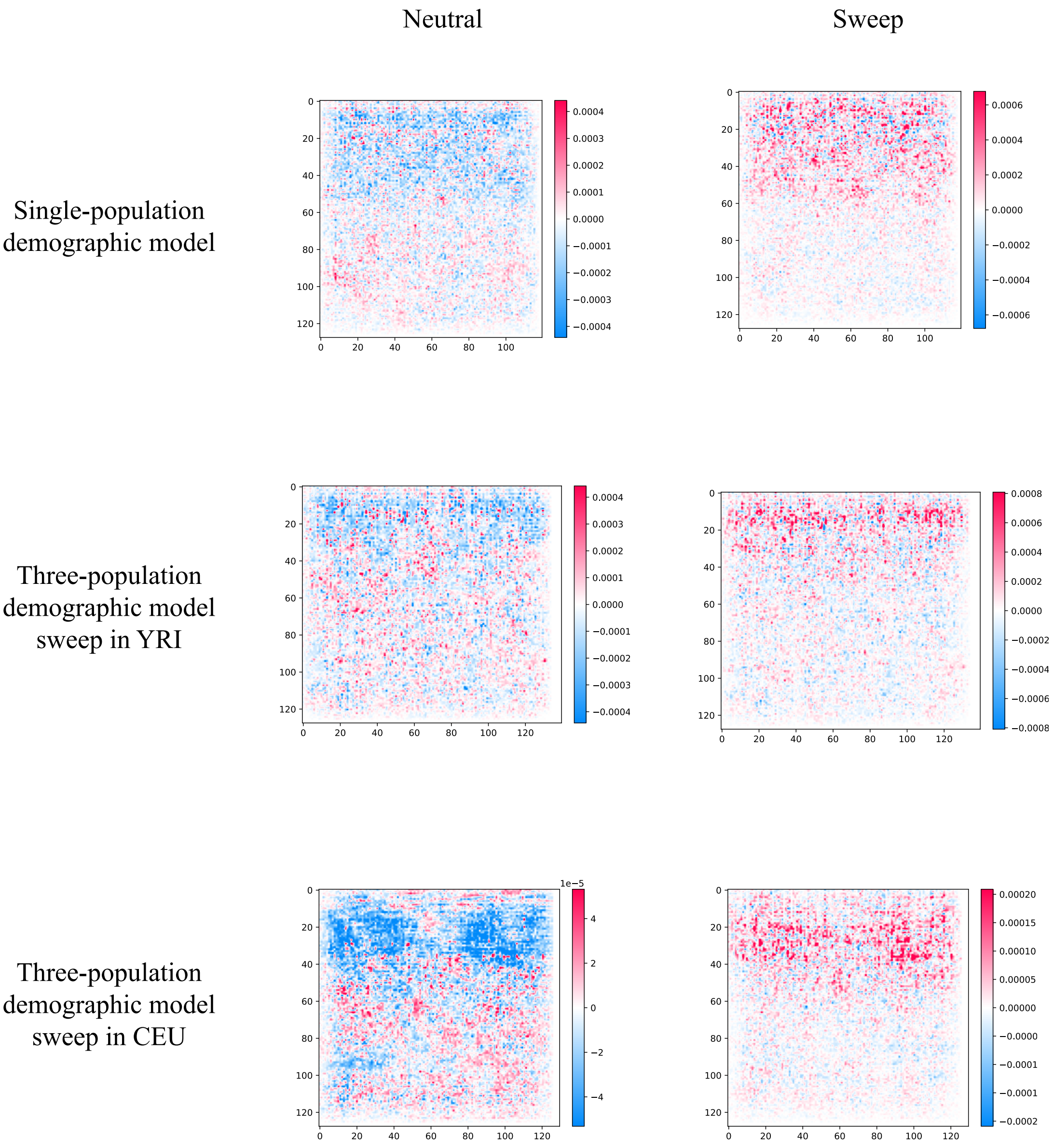

Figure L SHAP explanations for Imagene predictions with trimming for standardizing image width. To achieve uniform image width, we determined the minimum number of columns present across the simulations used for training, rounded down to the nearest multiple of 10, then trimmed all images to this width. We see similar behavior here as we see with image resizing (but not zero-padding): the CNN is attentive to the top of the image.

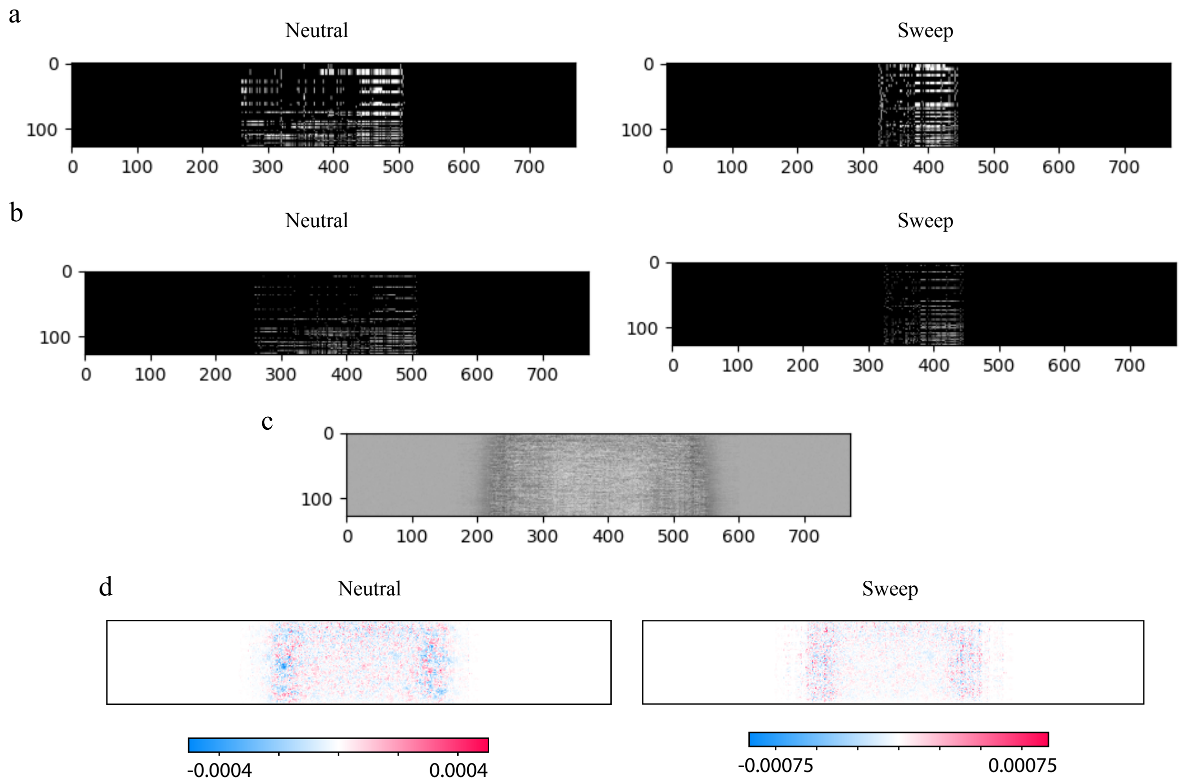

Figure M Full images corresponding to the cropped images in Figure 4.

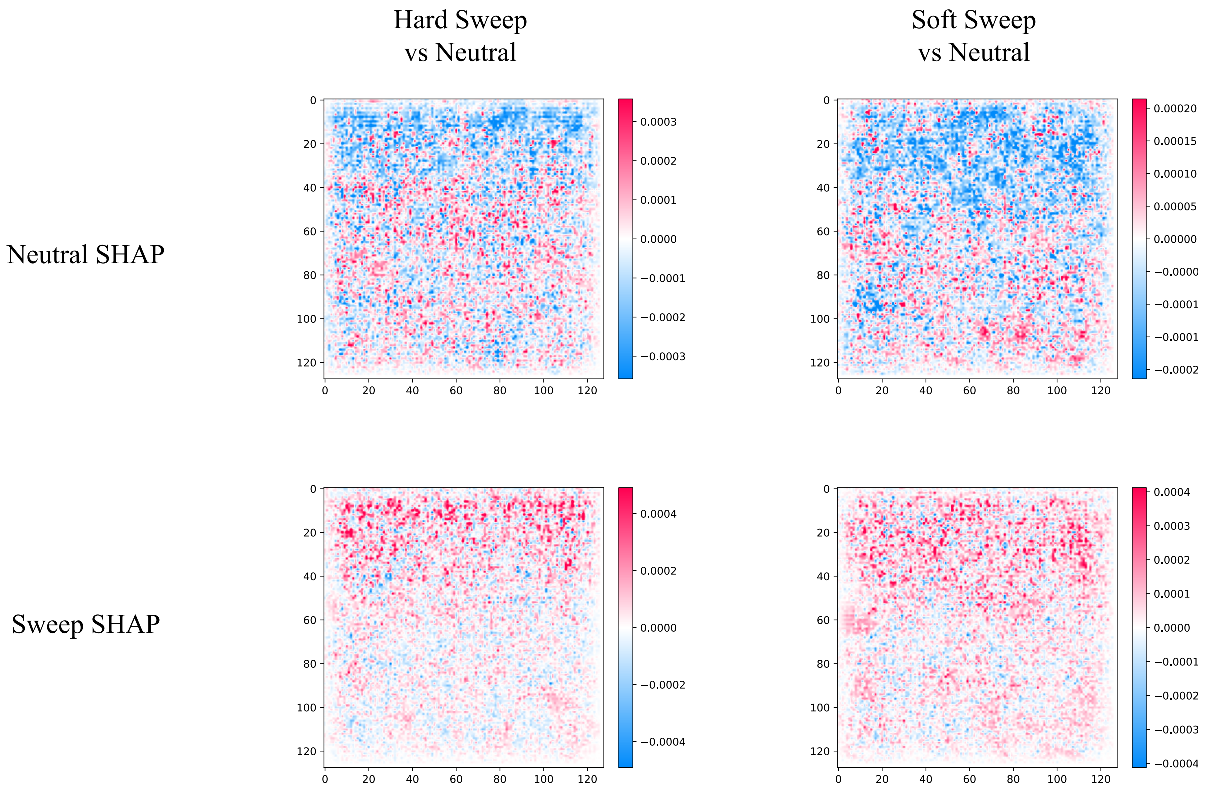

Figure N SHAP visualizations for Imagene trained to distinguish between hard/soft sweeps and neutrality We generated simulations from our single-population demographic model, introducing a sweep from standing variation in which a mutation present in 3% of the population acquires a selection coefficient of $s = 0.01$. On the right are average SHAP explanations for neutral and soft sweep simulations in training. On the left, for comparison, are the same explanations for Imagene trained on a hard sweep with the same selection coefficient. It is intriguing that the salient features seem to extend further down the image in the case of soft vs neutral, indicating that the model finds useful information from more haplotypes. We are hesitant to generalize these results, however, as our accuracies at the Hard/Soft task are quite low (Imagene: 60.7%, mini-CNN: 59.7%, Garud's H2/H1: 63.7%) and we have not optimized either our simulations or our model architectures for this task.

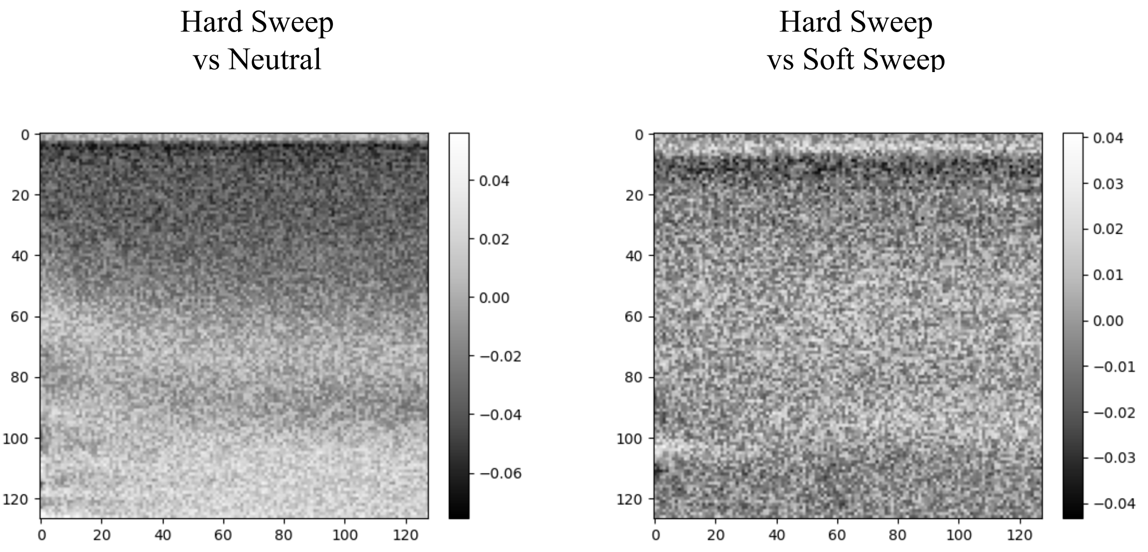

Figure O Visualizations of mini-CNN dense layer, comparing Hard vs Neutral and Hard vs Soft sweep classification. For the same soft sweep simulations referenced in Figure N, and our single-population hard sweeps with $s = 0.01$ we trained mini-CNN to classify between hard and soft sweeps. Looking at the dense layer when classifying Soft vs Hard, it is intriguing to see a pattern of dark pixels, corresponding to a classification of Hard, that is slightly further down the image than what we see when we classify Neutral vs Hard. The interpretation here would be that an absence of row-to-row differences in this lower stripe is the most salient signal to mini-CNN for a classification of Hard over Soft. This could make sense given that soft sweep results in a less robust signal of haplotype homozygosity. We are hesitant to generalize these results, however, as our accuracies at the Hard/Soft task are quite low (Imagene: 60.7%, mini-CNN: 59.7%, Garud's H2/H1: 63.7%) and we have not optimized either our simulations or our model architectures for this task.

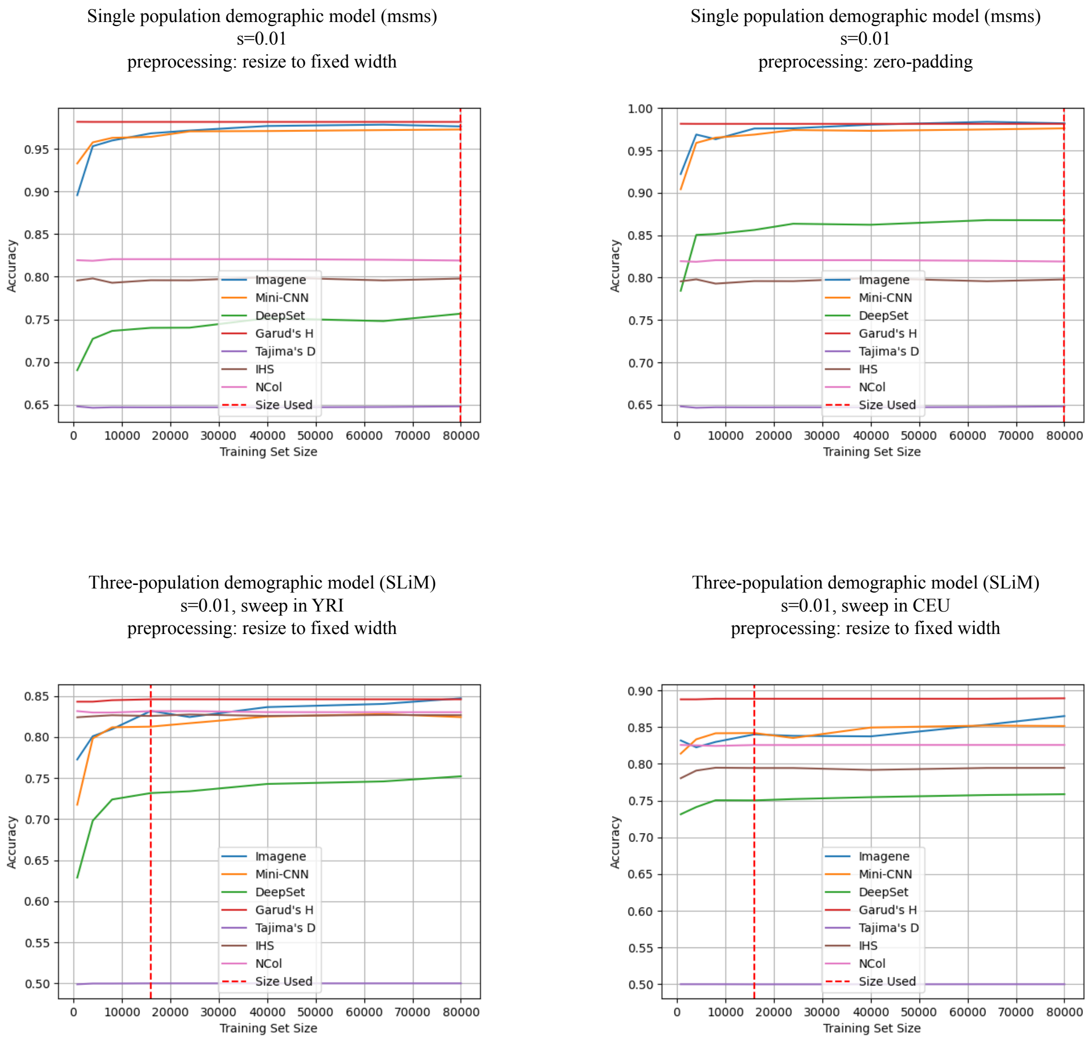

Figure P Sample Complexity Analysis. Analysis of performance with increasing training set size. Results are shown for the single-population and three-population demographic models for selection coefficient s=0.01, with the image-width standardization approaches denoted. Accuracy across a balanced test set is calculated across a range of training set sizes, for a range of CNN methods and summary statistics. Horizontal dotted red lines indicate the training set size used in the paper; we see in particular that for the more computationally intensive simulations (3 populations with forward simulator SLiM), training sets larger than 18,000 (with 2,000 held out for testing and validation) do not offer dramatic performance increases.

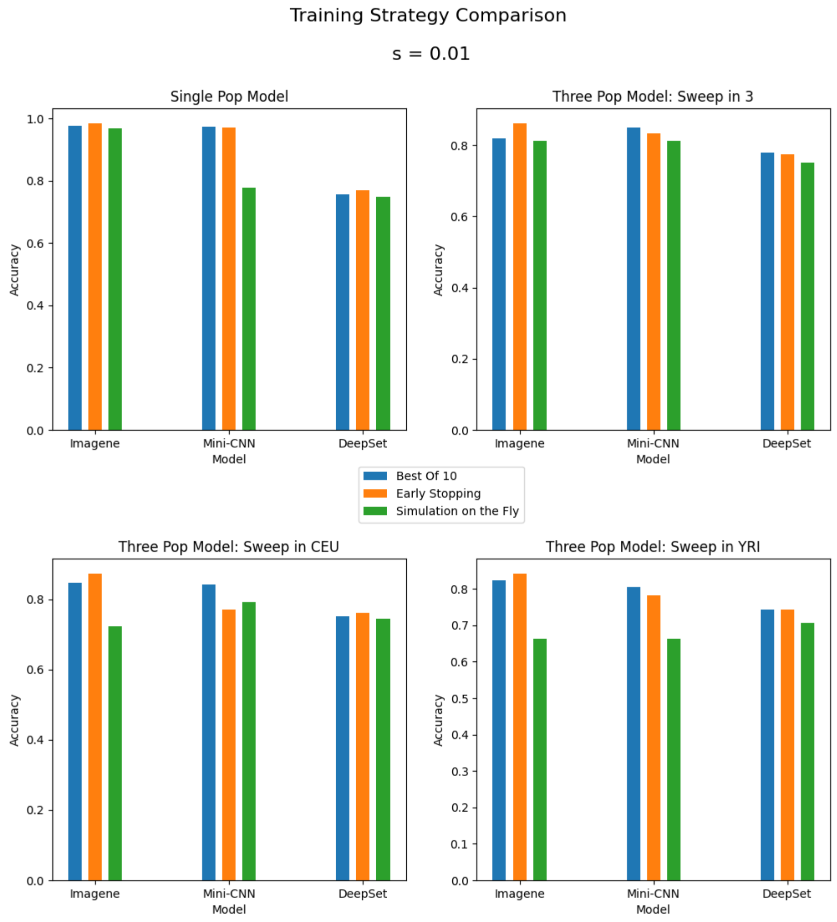

Figure Q Visualization of model performances across all demographic models and training strategies, for selection coefficient of 0.01 Each sub-plot corresponds to a different demographic model, the x-axis denotes the model type, the y-axis corresponds to the accuracy of the model on the balanced, held-out set, and the color of the bar corresponds to the training strategy. We refer to an epoch as a single training pass through the training dataset. The "Best of 10" training strategy trains the model 10 times for 2 epochs each, then selects the best performing model from the 10 trainings using the validation set. The "Early Stopping" strategy trains the model for 100 epochs, and utilizes early stopping to stop training whenever the validation accuracy fails to increase after 2 epochs of training. The "Simulation on the Fly" strategy follows the strategy used in Torada *et al.*[1], which trains the model for a single epoch, meaning that each training simulation is used only once. This approach may be viewed as simulating samples on the fly where the total number of simulated samples was decided upon prior to training. The early stopping and simulation on the fly trainings were restarted if the model failed to improve in accuracy after the first epoch. Across all the demographic models and selection coefficients, the range of accuracy differences between the early stopping and best of 10 strategies were $(-0.0599, 0.0445)$, $(-0.0725, -0.0019)$, and $(-0.097, 0.0137)$ for Imagene, Mini-CNN, and Deepset, respectively. The same range of accuracy differences between the simulation on the fly and best of 10 strategies were $(-0.1595, 0.0100)$, $(-0.1956, 0.0155)$, and $(-0.0490, -0.0078)$. We note that this implementation of "Simulation on the fly" may be at a disadvantage in this comparison because the additional epochs of the other training strategies result in more updates to the model during training; while it is possible to continue simulating novel data for simulation on the fly, our sample complexity analyses (see Figure P) indicate that we would likely see diminishing returns and would not expect this to result in much higher accuracy than we see with the other training strategies.

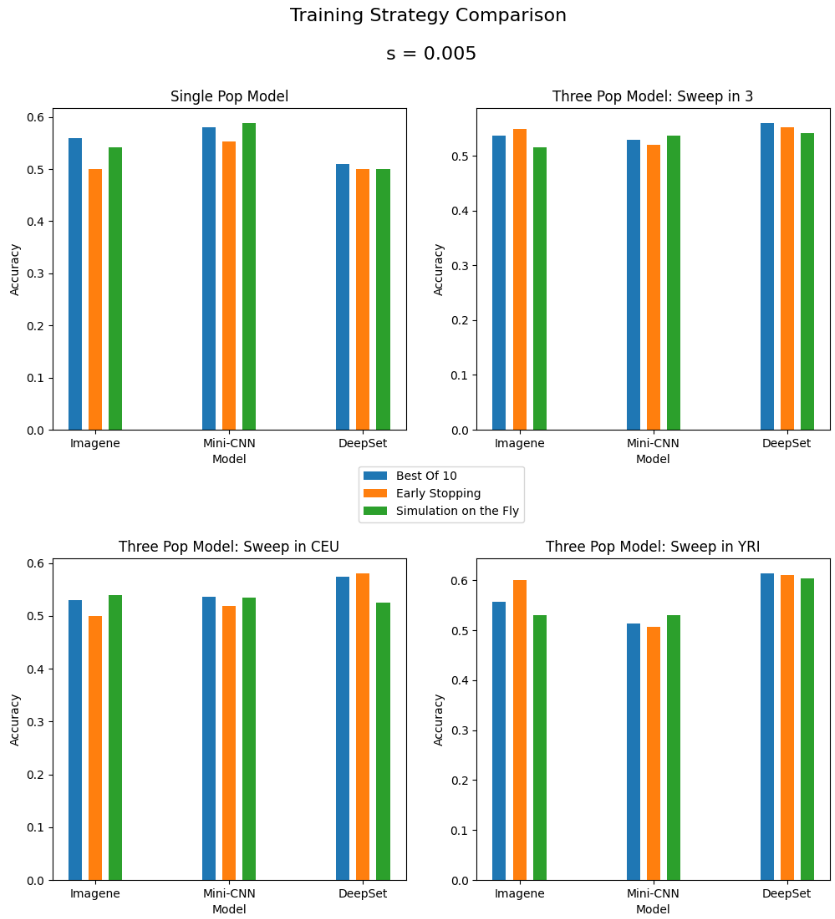

Figure R Visualization of model performances across all demographic models and training strategies, for selection coefficient of 0.005 Each sub-plot corresponds to a different demographic model, the x-axis denotes the model type, the y-axis corresponds to the accuracy of the model on the balanced, held-out set, and the color of the bar corresponds to the training strategy. We refer to an epoch as a single training pass through the training dataset. The "Best of 10" training strategy trains the model 10 times for 2 epochs each, then selects the best performing model from the 10 trainings using the validation set. The "Early Stopping" strategy trains the model for 100 epochs, and utilizes early stopping to stop training whenever the validation accuracy fails to increase after 2 epochs of training. The "Simulation on the Fly" strategy follows the strategy used in Torada *et al.*[1], which trains the model for a single epoch, meaning that each training simulation is used only once. This approach may be viewed as simulating samples on the fly where the total number of simulated samples was decided upon prior to training. The early stopping and simulation on the fly trainings were restarted if the model failed to improve in accuracy after the first epoch. Across all the demographic models and selection coefficients, the range of accuracy differences between the early stopping and best of 10 strategies were $(-0.0599, 0.0445)$, $(-0.0725, -0.0019)$, and $(-0.097, 0.0137)$ for Imagene, Mini-CNN, and Deepset, respectively. The same range of accuracy differences between the simulation on the fly and best of 10 strategies were $(-0.1595, 0.0100)$, $(-0.1956, 0.0155)$, and $(-0.0490, -0.0078)$. We note that this implementation of "Simulation on the fly" may be at a disadvantage in this comparison because the additional epochs of the other training strategies result in more updates to the model during training; while it is possible to continue simulating novel data for simulation on the fly, our sample complexity analyses (see Figure P) indicate that we would likely see diminishing returns and would not expect this to result in much higher accuracy than we see with the other training strategies.

References

[1] Luis Torada et al. "ImaGene: a convolutional neural network to quantify natural selection from genomic data". In: *BMC Bioinformatics* 20 (2019). DOI: https://doi.org/10.1186/s12859-019-2927-x. URL: https://bmcbioinformatics.biomedcentral.com/articles/10.1186/s12859-019-2927-x.
