# Supplementary material for: On convolutional neural networks for selection inference: Revealing the effect of preprocessing on model learning and the capacity to discover novel patterns
Source: PLoS Comput Biol. 2023 Nov 27;19(11):e1010979. doi: 10.1371/journal.pcbi.1010979 (PMC10703409; doi:10.1371/journal.pcbi.1010979)
Supplement: Attachment — Submitted filename: PCOMPBIOL_review.docx [file pcbi.1010979.s003.docx]

This manuscript, by Ryan Cecil and Lauren Sugden, presents a set of simulation experiments that aim to probe the behavior of deep learning models under the hood. Specifically, Cecil & Sugden interpret the convolution filters and saliency maps to uncover the features in the genotypic matrices used by the CNNs to classify selective sweeps. The result suggests that common preprocessing procedures of the genotypic matrices may introduce artificial “short-cut” features exploited by the deep learning model, causing the model to mimic the behavior of haplotype summary statistics such as H1. The reliance on these artificial features suggests that the potential of CNNs learning more complex features from the data may not be fully utilized in the current applications of these deep learning models.

Although deep learning models have become more popular among population genetic researchers as a “black box” estimator a variety of inference problems, not much effort has been put into interpreting the inner workings of these models. This study marks one of the early attempts to understand how deep learning models make predictions specifically in the context of selective sweep inference. I believe this work is of great interest to researchers working on deep learning method development for popgen problems.

While the authors have identified an important issue and defined the scope of this work well within a broader context, the manuscript could benefit from several additional analyses to substantiate or qualify its main conclusions.

Major comments

- It seems like, from descriptions in the method section (l.378-380, l.391-392), that all analyses in this work focused on *hard* sweep inference. It is perfectly reasonable to start with the simplest case of a sweep that leaves clear genomic signatures. However, it remains to be seen whether simple summary statistics (H12, H2/H1 etc.) can match the performance of CNNs in harder cases such as *soft* sweeps (or distinguishing different modes of sweeps). Ideally, this hypothesis should be tested with another set of similar experiments using soft sweep simulations. Multiple dominant haplotypes (created by soft sweeps) might look very interesting on the saliency map. Otherwise, without experimental evidence of the more complex scenarios, the limitation of the study should be acknowledged. While the finding that H1 can perform surprisingly well is insightful, it should be carefully interpreted given the experiment is only done for hard sweeps.
- Regarding the 1^st^ major preprocessing procedure explored in this study -- **sorting**: It is really cool to see the authors compare the performance of the CNNs with that of permutation-invariant networks (l.239-264), and along with it interesting discussion about what row-sorting does to create “short-cuts” for the CNNs. While it is a major topic in this paper, the authors mostly relied on observations from previous studies to reason about the effect of row sorting on model performance (l. 72-73, 325-335). I think this work has the potential to be a comprehensive study that systematically examines the behavior of CNNs with and without sorting. Using saliency maps that are already heavily featured in the study, one could perhaps examine what features a CNN might rely on when the haplotypes are unsorted and the “short-cut” features are missing. Another incentive to replicate the comparison between sorted and unsorted models is that although both Flagel et al. and Torada et al. performed the experiment, they used different metrics and the results are not directly comparable. On top of the insights gained via saliency maps, an interesting hypothesis that could be tested is how sorting differentially affects the performance of 1D (Flagel et al. architecture) vs. 2D (Torada et al.) conv kernels. The author briefly touched on this topic (l.274-276), but it could be a very interesting point to expand on. One intuition is that 1D kernels could be more robust when haplotypes are unsorted since they act on whole columns and are more suited to discover global features, whereas 2D kernels that extract local features are more prone to the effects of sorting. The paper would greatly benefit from additional empirical evidence to flush out this heavily discussed topic on sorting.
- Re. 2^nd^ major preprocessing procedure -- **padding/resizing**: While there is an important lesson to be learned about short-cut features that create very strong signals, it feels more of a preventable artifact (at least compared to the sorting problem). To me the genotypic matrix resizing problem mirrors the window size choice problem in calculating summary statistics. In the original Garud et al. paper, H statistics were calculated in 400-SNP windows, based on neutral expectation of LD decay in particular population of interest (which roughly correspond to a ~10kb window). This should be something easy to do for standardizing the size of genotypic matrices (as the authors alluded to on l. 68), by allowing varying genomic ranges in applications to real data. This does require simulation of larger genomic segments for training such that there are always enough SNPs in a segment, which increases the computational footprint. However, this is a viable strategy that addresses the issues mentioned on l.232-237 (this does not blow up the input dim, while retaining haptype structure) as well as that suggested on l.321-324 (when there are sufficient number of SNPs retained from a simulated region, there is very little information in the SNPs thrown away because of LD decay). It would be interesting to evaluate the performance of this strategy, or at least offer it as a possible remedy for the problem. Another important aspect relating to the padding issue, which is unexplored in this paper, is that incorporating inter-SNP distances as a feature vector (i.e. Flagel et al. branched architecture) might have a significant effect on the performance of CNN models without padding. Intuitively, assuming uniform mutation and recombination rate, the inter-SNP distances should contain very similar information as the “nCols” statistics used in the study. Perhaps it’s worth acknowledging this limitation of the study but explain how it belongs to future work since it can be very hard to interpret a branched architecture.
- l. 433-434: I am a little concerned about the training procedure. Is there a particular reason not to use an “early-stopping” strategy by monitoring validation loss on a validation set, but fix training to just 2 epochs? Both Flagel et al. and ImaGene (Torada et al.) trained their models with “early-stopping,” with ImaGene going even further with simulation-on-the-fly. 2 epochs might be enough for the mini CNN, but the larger models could require more epochs. This can be a major issue because all the performance comparisons rest on the NN being properly trained.

Minor comments

- l. 18: “Korfmann et al. 2023 *Genome Biol. Evol.*” could be a highly relevant recent review paper to mention here.
- l. 34: It’s really great that the authors took the effort to compile the details of these methods and made them available.
- l. 35: It might be worthwhile to clarify here that the “testing data” are simulated, and mostly under the same models that generated the training data.
- l.79-82: Related to a previous comment, this might be where you could quickly acknowledge that interpreting a branched CNN such as the one in Flagel et al. could be very challenging.
- l.141-142; Table 1: Do you have a way to come up with some uncertainty measure of the performance? Not sure if it is very meaningful to say that H1 *outperforms*, without some sort of estimate of how the performance varies from run to run, especially when the difference is small. It is striking, though, that H1 is on par with the CNNs.
- l.170-172: I am a bit lost here. I thought the performances reported in Table S1 are from simulations under the single-population model?
- l.185: `scikit-image` is designed for actual images and visual consistency, it could be a reasonable suspicion that it would not work well on genotypic matrices and therefore a poor baseline for the comparison. Perhaps a very brief intuition/summary of what the method does (maybe in the method section, l.403-405) to assure the reader that it performs reasonable operations on genotypic matrices (e.g. there is no anti-aliasing or other distortions) would help establish it as a valid baseline.
- l.193, l.225: Do you mean S2 Table instead of S1?
- l.316-319: Don’t mutation and recombination rate variations equally affect sum stats like H?
- l.319-321: This could possibly be argued as a strength for ML method, which can use simulations to cover complex scenarios and possibly discover robust features. This is something sum stats cannot do.
- l.324: Typo? What is “[Otherwise]”?
- S1&S2 tables: The presentation of model accuracies is hard to follow, especially considering these results support the key conclusions of this paper. One possibility could be using line-and-point plots (e.g. <https://seaborn.pydata.org/tutorial/categorical.html#point-plots>) to make the trend stand out. Also, it might help to include the plot as one of the main figures, since these results are central to the narrative.
- A top-level README file could help the readers navigating through your GitHub repo (accessed 03/21/2023).
