# Supplementary material for: On convolutional neural networks for selection inference: Revealing the effect of preprocessing on model learning and the capacity to discover novel patterns
Source: PLoS Comput Biol. 2023 Nov 27;19(11):e1010979. doi: 10.1371/journal.pcbi.1010979 (PMC10703409; doi:10.1371/journal.pcbi.1010979)
Supplement: Attachment — Submitted filename: response_to_reviewers.docx [file pcbi.1010979.s004.docx]

Dear Mr. Cecil,

Thank you very much for submitting your manuscript "On convolutional neural networks for selection inference: revealing the lurking role of preprocessing, and the surprising effectiveness of summary statistics" for consideration at PLOS Computational Biology.

As with all papers reviewed by the journal, your manuscript was reviewed by members of the editorial board and by several independent reviewers. In light of the reviews (below this email), we would like to invite the resubmission of a significantly-revised version that takes into account the reviewers' comments.

We cannot make any decision about publication until we have seen the revised manuscript and your response to the reviewers' comments. Your revised manuscript is also likely to be sent to reviewers for further evaluation.

When you are ready to resubmit, please upload the following:

[1] A letter containing a detailed list of your responses to the review comments and a description of the changes you have made in the manuscript. Please note while forming your response, if your article is accepted, you may have the opportunity to make the peer review history publicly available. The record will include editor decision letters (with reviews) and your responses to reviewer comments. If eligible, we will contact you to opt in or out.

[2] Two versions of the revised manuscript: one with either highlights or tracked changes denoting where the text has been changed; the other a clean version (uploaded as the manuscript file).

Important additional instructions are given below your reviewer comments.

Please prepare and submit your revised manuscript within 60 days. If you anticipate any delay, please let us know the expected resubmission date by replying to this email. Please note that revised manuscripts received after the 60-day due date may require evaluation and peer review similar to newly submitted manuscripts.

Thank you again for your submission. We hope that our editorial process has been constructive so far, and we welcome your feedback at any time. Please don't hesitate to contact us if you have any questions or comments.

Sincerely,

Piero Fariselli
Academic Editor
PLOS Computational Biology

James O'Dwyer
Section Editor
PLOS Computational Biology

***********************

We are grateful to the editor for the invitation to resubmit, and for directing our paper to these three reviewers. They have made excellent suggestions regarding both additional analyses and rhetorical points that we believe strengthen the narrative significantly. Additional analyses now include analysis of models on unsorted data, models trained on larger images with more haplotypes, using trimming as an alternative image-width standardization approach, and training with different stopping criteria to ensure our models are properly trained. We have also included some preliminary analyses of soft sweeps. Rhetorically, we have adjusted how we discuss our findings surrounding zero-padding to add nuance and to acknowledge that the number of segregating sites in a window is a genuine signature of a sweep, and we provide concrete suggestions for those who wish to use this method to help them avoid overfitting on training data. Together with many other rhetorical changes and additions, additional figure panels, and additional supplementary figures and tables, we think that the manuscript is significantly strengthened, and we are proud to resubmit it for consideration by the editor and reviewers.

Reviewer's Responses to Questions

**Comments to the Authors:
Please note here if the review is uploaded as an attachment.**

Reviewer #1: The review is uploaded as an attachment.

This manuscript, by Ryan Cecil and Lauren Sugden, presents a set of simulation experiments that aim to probe the behavior of deep learning models under the hood. Specifically, Cecil & Sugden interpret the convolution filters and saliency maps to uncover the features in the genotypic matrices used by the CNNs to classify selective sweeps. The result suggests that common preprocessing procedures of the genotypic matrices may introduce artificial “short-cut” features exploited by the deep learning model, causing the model to mimic the behavior of haplotype summary statistics such as H1. The reliance on these artificial features suggests that the potential of CNNs learning more complex features from the data may not be fully utilized in the current applications of these deep learning models.

Although deep learning models have become more popular among population genetic researchers as a “black box” estimator a variety of inference problems, not much effort has been put into interpreting the inner workings of these models. This study marks one of the early attempts to understand how deep learning models make predictions specifically in the context of selective sweep inference. I believe this work is of great interest to researchers working on deep learning method development for popgen problems.

While the authors have identified an important issue and defined the scope of this work well within a broader context, the manuscript could benefit from several additional analyses to substantiate or qualify its main conclusions.

We would like to thank this reviewer for their thorough and thoughtful review, and for the suggestions they have made for strengthening the manuscript. We have carried out the suggested additional analyses, including simulating soft sweeps and investigating other methods of training and preprocessing. As a result, we think our results are much more robust, and our discussion of the current state of CNNs in the field is more complete. Please see our responses to each comment below. Note that excerpts contain line numbers for easy reference, and new text appears in red.

Major comments

- It seems like, from descriptions in the method section (l.378-380, l.391-392), that all analyses in this work focused on hard sweep inference. It is perfectly reasonable to start with the simplest case of a sweep that leaves clear genomic signatures. However, it remains to be seen whether simple summary statistics (H12, H2/H1 etc.) can match the performance of CNNs in harder cases such as soft sweeps (or distinguishing different modes of sweeps). Ideally, this hypothesis should be tested with another set of similar experiments using soft sweep simulations. Multiple dominant haplotypes (created by soft sweeps) might look very interesting on the saliency map. Otherwise, without experimental evidence of the more complex scenarios, the limitation of the study should be acknowledged. While the finding that H1 can perform surprisingly well is insightful, it should be carefully interpreted given the experiment is only done for hard sweeps.

We agree that our results need to be accurately contextualized, and we have added text to a few places to make clear that our results are not generalizable beyond the hard sweep context. In particular, we have added the following paragraph to our introduction:

“We note, finally, that our analyses here are restricted only to the case of recent hard 60

selective sweeps. CNNs have been shown to be effective across a wide range of 61

applications even within evolutionary biology, including for detecting introgression[18], 62

soft sweeps[28], and balancing selection[25]. We hope that this work can serve as a case 63

study for how these other CNNs might be explored and mined for insights. We do not 64

intend to generalize our particular results to those scenarios.” 65

At the same time, we agree with the reviewer that it would be interesting to see this analysis applied to soft sweeps. To this end, we have generated a set of soft sweeps using the same single-population model with which we generated hard sweeps. Specifically, we simulated sweeps from standing variation, in which the beneficial allele is present in 3% of the population prior to the onset of selective pressure. We trained the Imagene model to classify between soft sweeps and neutral simulations, and confirmed that we are still able to pare the model down to mini-CNN and maintain similar performance. We see similar correlation patterns among the CNNs and summary statistics here that we see with hard sweeps, and the SHAP diagrams also appear to show attention to the top of the image (S14 Fig). At a glance, it appears that the area of interest for soft sweep detection might extend further downward in the image compared to the area of interest for hard sweep detection, but we are hesitant to make any general conclusions here. We also trained our CNNs to classify between hard and soft sweeps, and have included dense layer visualizations for the trained model, which show an intriguing signature of paying closest attention to rows close to but not at the top of the image (S15 Fig). We have chosen to discuss these results in the Discussion, because we believe they are too preliminary and deserve closer attention that is outside the scope of this manuscript. The text is excerpted below, and we note that our supplementary figure captions emphasize that these are preliminary and should not be taken to be definitive without more extensive simulations and testing.

“On the other hand, all of the models we implemented lose significant 325

performance when selection coefficients become weaker, especially in the context of 326

more complex demographic backgrounds. A similar pattern might be expected with 327

older sweeps, or selection over a much longer timescale, which might necessitate 328

different summary statistics, and would require different training simulations for the 329

CNNs. Another example in this vein is distinguishing between soft and hard sweeps: a 330

few preliminary experiments training Imagene and mini-CNN for this task resulted in 331

fairly low accuracy (60.7% and 59.7% respectively), but did provide a couple of 332

intriguing explanatory visualizations that may point in potentially fruitful directions for 333

future research (S14 Fig, S15 Fig)...” 334

[...]

“...In all of these cases, the additional 347

complexity of the CNNs may well be beneficial, especially in areas with less robust 348

suites of summary statistics than the hard sweep case. Ideally, by examining these 349

CNNs, insight could be gained that might result in more easily-applied tools in order to 350

make them more broadly available; we hope that this study provides some inspiration 351

for such investigations, which can dovetail with cutting-edge interpretive and 352

explanatory tools as they emerge.” 353

- Regarding the 1st major preprocessing procedure explored in this study -- sorting: It is really cool to see the authors compare the performance of the CNNs with that of permutation-invariant networks (l.239-264), and along with it interesting discussion about what row-sorting does to create “short-cuts” for the CNNs. While it is a major topic in this paper, the authors mostly relied on observations from previous studies to reason about the effect of row sorting on model performance (l. 72-73, 325-335). I think this work has the potential to be a comprehensive study that systematically examines the behavior of CNNs with and without sorting. Using saliency maps that are already heavily featured in the study, one could perhaps examine what features a CNN might rely on when the haplotypes are unsorted and the “short-cut” features are missing. Another incentive to replicate the comparison between sorted and unsorted models is that although both Flagel et al. and Torada et al. performed the experiment, they used different metrics and the results are not directly comparable. On top of the insights gained via saliency maps, an interesting hypothesis that could be tested is how sorting differentially affects the performance of 1D (Flagel et al. architecture) vs. 2D (Torada et al.) conv kernels. The author briefly touched on this topic (l.274-276), but it could be a very interesting point to expand on. One intuition is that 1D kernels could be more robust when haplotypes are unsorted since they act on whole columns and are more suited to discover global features, whereas 2D kernels that extract local features are more prone to the effects of sorting. The paper would greatly benefit from additional empirical evidence to flush out this heavily discussed topic on sorting.

We appreciate these suggestions, and we have added our own analysis using unsorted data. Similar to what others have found, we show that the performance of both Imagene and mini-CNN are substantially reduced relative to their performance on row-sorted data, although they still perform better than chance. This makes intuitive sense, since in the presence of selection, the larger number of similar haplotypes will result in an elevated chance of row-to-row similarity even in unsorted data. Interestingly, we find that Imagene’s architecture cannot be reduced all the way down to mini-CNN the same way it can be for sorted data (see S2 Table). In particular, to maintain the performance of the full Imagene model, four 3x3 kernels are necessary. To the reviewer’s point above, this indicates that a lack of sorting makes 1D kernels much less effective compared to 2D kernels. Since we cannot reduce the complexity down to mini-CNN, we cannot use the dense layer of mini-CNN as a particularly useful visualization, but we can use SHAP values with the Imagene model to try to get a glimpse of what the model is learning (see S2 Fig). Perhaps as expected, these diagrams do not show any discernible patterns (importantly, as a sanity check, they do not show the particular top-heavy pattern we see in the presence of row sorting). We hope that as the cutting-edge of CNN interpretability and explainability advances, there will be opportunities to delve deeper into these observations, but we would argue that they are currently beyond the scope of this manuscript. The following is an excerpt from the Results section that discusses these results:

“As a control, we include SHAP diagrams for Imagene trained on unsorted data in S2 134

Fig; when the row-sorting is not included as a preprocessing step, this pattern 135

disappears and it becomes difficult to discern any relevant patterns. It is notable that 136

despite this, Imagene still performs better than chance, with an accuracy of around 75%. 137

A possible explanation for this is that there is still an elevated chance of row-to-row 138

similarity after a sweep, even if these similarities are not deliberately gathered at the 139

top of the image. Interestingly, mini-CNN does not perform as well as Imagene on 140

unsorted data; in particular, while the number of layers can be reduced, we find that 141

four 3x3 kernels are necessary to maintain the performance of Imagene (see S2 Table). 142

We note that our goal here is to understand the effects of common pre-processing steps, 143

and to learn from the top-performing models, all of which rely on the images being 144

row-sorted. As such, we leave further analysis of this result to future work, which will 145

certainly benefit from advances in interpretive and explanatory tools”. 146

- Re. 2nd major preprocessing procedure -- padding/resizing: While there is an important lesson to be learned about short-cut features that create very strong signals, it feels more of a preventable artifact (at least compared to the sorting problem). To me the genotypic matrix resizing problem mirrors the window size choice problem in calculating summary statistics. In the original Garud et al. paper, H statistics were calculated in 400-SNP windows, based on neutral expectation of LD decay in particular population of interest (which roughly correspond to a ~10kb window). This should be something easy to do for standardizing the size of genotypic matrices (as the authors alluded to on l. 68), by allowing varying genomic ranges in applications to real data. This does require simulation of larger genomic segments for training such that there are always enough SNPs in a segment, which increases the computational footprint. However, this is a viable strategy that addresses the issues mentioned on l.232-237 (this does not blow up the input dim, while retaining haptype structure) as well as that suggested on l.321-324 (when there are sufficient number of SNPs retained from a simulated region, there is very little information in the SNPs thrown away because of LD decay). It would be interesting to evaluate the performance of this strategy, or at least offer it as a possible remedy for the problem.

We agree with the reviewer that this scheme, in which larger simulations are pared down (or “trimmed”) to a common width, provides a viable strategy for researchers who do not wish to use resizing or padding (we note here that our discussion of padding has changed as well, in response to reviewer #3), and that the manuscript would benefit from an actual demonstration of this strategy. We have implemented a version of this, in which we allow for varying genome spans across haplotype matrices by trimming simulated matrices down to a common number of SNPs. Since our original simulations are already larger than the predicted footprint of the sweep (80kb vs ~50kb; we refer to this in the following paragraph/excerpt), we have used these simulations as a starting point, but we acknowledge in the text that even larger simulations might lead to better performance. The details of this implementation have been added to Materials and Methods, accuracy values are in S3 Table, SHAP visualizations are now in S12 Fig, and the results are discussed in the Results section newly titled Effects of image-width standardization on CNN learning strategies, excerpted here:

“We also implemented a trimming strategy (see Materials and Methods), with results 264

shown in S3 Table. With this image-width standardization strategy, we see similar 265

results as with image resizing. In particular, mini-CNN performs on par with Imagene, 266

with Garud’s H continuing to outperform both. This method of standardization again 267

makes the row-sorting the most salient signal, as interpreted by SHAP diagrams (see S12 268

Fig). We do see a decrease in performance relative to image resizing, which we attribute 269

to the fact that we are trimming out potentially useful columns from some simulations. 270

We note that this could likely be ameliorated by starting with even larger simulations to 271

ensure that the minimum number of SNPs per simulation is above a desired threshold.” 272

In addition, we appreciate the reviewer’s focus on the choice of window size, and the ability to build this into the experimental design in a deliberate way. We think this is an important point, especially as we compare CNNs with summary statistics, some of which we might expect to be very sensitive to this. We have added the following paragraph to the Results section “Comparison to Summary Statistics”:

“We note here that in evaluating the performance of Garud’s H1, along with other 174

window-based summary statistics, it is important to consider the window size as it 175

relates to the expected sweep footprint. Following Harris et al.[22], and using our 176

single-population demographic model parameters, we estimate a sweep footprint on the 177

order of 50kb for s = 0.01. Our simulations, which span 80kb, should therefore 178

represent a realm that allows for good, if not optimal, summary statistic performance. 179

With a much larger window size, their performance might be expected to decline, while 180

we may expect the CNNs to maintain their performance and learn to ignore columns 181

outside of the sweep footprint.” 182

Another important aspect relating to the padding issue, which is unexplored in this paper, is that incorporating inter-SNP distances as a feature vector (i.e. Flagel et al. branched architecture) might have a significant effect on the performance of CNN models without padding. Intuitively, assuming uniform mutation and recombination rate, the inter-SNP distances should contain very similar information as the “nCols” statistics used in the study. Perhaps it’s worth acknowledging this limitation of the study but explain how it belongs to future work since it can be very hard to interpret a branched architecture.

We appreciate this point, and we have included text to address it in two places. The first is in the Results section, as recommended by this reviewer in minor comment #4 (see below for excerpt). In addition, we have added some language to the discussion where we discuss limitations of this study (new text in red; note that there are other changes in this section in response to other reviewer comments):

“This raises the possibility that more complex, tailored models could detect signatures 335

that are as-yet unobserved, given the right architecture and simulation scheme. In 336

particular, we note that the supervised nature of CNNs allows for these models to be 337

trained on complex demographies and evolutionary scenarios to approximate real-world 338

data and draw out nuanced signals in ways that summary statistics may not be able to. 339

To this point, CNNs have been shown to be effective in other population genetics 340

applications including identifying recombination hotspots[3] and adaptive 341

introgression[18], and distinguishing between soft sweeps and balancing selection[25], 342

none of which we explore here. We also note that there are more complex architectures 343

that exist in the literature, including a branched architecture that takes in positional 344

data in addition to the haplotype image[14], potentially allowing for the model to learn 345

more nuanced signatures. This additional model complexity makes interpretation more 346

difficult, so we have not attempted it here. In all of these cases, the additional 347

complexity of the CNNs may well be beneficial, especially in areas with less robust 348

suites of summary statistics than the hard sweep case. Ideally, by examining these 349

CNNs, insight could be gained that might result in more easily-applied tools in order to 350

make them more broadly available; we hope that this study provides some inspiration 351

for such investigations, which can dovetail with cutting-edge interpretive and 352

explanatory tools as they emerge.” 353

- l. 433-434: I am a little concerned about the training procedure. Is there a particular reason not to use an “early-stopping” strategy by monitoring validation loss on a validation set, but fix training to just 2 epochs? Both Flagel et al. and ImaGene (Torada et al.) trained their models with “early-stopping,” with ImaGene going even further with simulation-on-the-fly. 2 epochs might be enough for the mini CNN, but the larger models could require more epochs. This can be a major issue because all the performance comparisons rest on the NN being properly trained.

We understand the reviewer’s concern here, and we have undertaken an experiment to ensure that our models are properly trained. In particular, for each of our CNN models, on each demographic model and with each selection coefficient, we compare the performance of our training scheme with both early stopping, and simulation-on-the-fly. We find that no one scheme works the best across the board, and in general, our scheme performs on par with early stopping, while both generally outperform simulation-on-the-fly. We have added new supplementary figures S17 Fig and S18 Fig, referenced in Materials and Methods, that shows these accuracy values across demographic models and selection coefficients. In the cases where early stopping slightly outperforms our training scheme, the major takeaways concerning the relative performance with Garud’s H is unchanged.

Minor comments

- l. 18: “Korfmann et al. 2023 Genome Biol. Evol.” could be a highly relevant recent review paper to mention here.

We thank the reviewer for bringing our attention to this paper. It is now included as a reference in the relevant place.

- l. 34: It’s really great that the authors took the effort to compile the details of these methods and made them available.

We appreciate the reviewer’s appreciation! We do think this transparency is an important aspect of the manuscript.

- l. 35: It might be worthwhile to clarify here that the “testing data” are simulated, and mostly under the same models that generated the training data.

We agree that this is an important point. We have updated the relevant sentence to read as follows:

“While all methods report high accuracy on simulated testing data (typically generated 38

in the same way as the training data), what is as yet unexplored is how these models 39

work, what preprocessing decisions they might be sensitive to, and what insights they 40

might be able to provide to our current theory.” 41

- l.79-82: Related to a previous comment, this might be where you could quickly acknowledge that interpreting a branched CNN such as the one in Flagel et al. could be very challenging.

We thank the reviewer for helping us find a suitable place for this! We have added the following text to this section:

“Interestingly, the choice of CNN architecture varies 87

widely across these works. The number of kernel dimensions, kernels, and convolutional 88

layers alone range from 1D-2D, 4-256, and 1-4, respectively. Some models employ a 89

branched architecture, allowing for additional inputs beyond the haplotype image [14]; 90

since this presents an additional challenge for interpretation, we do not analyze 91

branched architectures here. Together with varying architecture, models in the 92

literature also vary in terms of the chosen demographic model and simulation software; 93

altogether this makes direct comparison between the approaches tricky. A close look 94

into how these CNN models make their predictions would aid in this regard.” 95

We have also added text to the Discussion, as referenced above in response to the second-to-last major comment.

- l.141-142; Table 1: Do you have a way to come up with some uncertainty measure of the performance? Not sure if it is very meaningful to say that H1 *outperforms*, without some sort of estimate of how the performance varies from run to run, especially when the difference is small. It is striking, though, that H1 is on par with the CNNs.

We can provide a measure of uncertainty with a standard error of the proportion (with our very large sample sizes, this is well-approximated by $\sqrt{\frac{\hat{p}(1-\hat{p})}{n}}$ ). Using these, we estimate 95% confidence intervals and add them as error bars in multiple places, including the new Figure 1 panel that compares Imagene and mini-CNN performance. In addition, we have added bar plots with error bars in new supplementary figures S4 Fig And S5 Fig for visualizing performance of additional models across additional demographic models and selection coefficients, and we provide bounds on standard error in the caption for Table 1. Across the board, these measures of uncertainty are very small, with a maximum standard error of ~1.1\% across all experiments.

- l.170-172: I am a bit lost here. I thought the performances reported in Table S1 are from simulations under the single-population model?

We appreciate the reviewer for catching this. This was indeed a mistake, and it has been removed.

- l.185: `scikit-image` is designed for actual images and visual consistency, it could be a reasonable suspicion that it would not work well on genotypic matrices and therefore a poor baseline for the comparison. Perhaps a very brief intuition/summary of what the method does (maybe in the method section, l.403-405) to assure the reader that it performs reasonable operations on genotypic matrices (e.g. there is no anti-aliasing or other distortions) would help establish it as a valid baseline.

We thank the reviewer for this suggestion. We agree that image resizing algorithms are not necessarily perfectly suited for binary genetic data. We note that our expanded analysis of other approaches (both zero-padding and trimming) hopefully provides some robustness here. We chose this specific resizing algorithm as our first method to follow the pre-processing steps used in Torada *et al*. in order to keep mini-CNN consistent with Imagene as published. It does implement anti-aliasing, however, we do not expect this to have a large impact on the performance of the models that utilize row-sorting. It may actually help to better preserve the sorted blocks within the image. We would expect the biggest impact to be on the unsorted images, where the features may look like noise in some locations. We have added discussion that the models appear to perform differently on sorted data, and may be looking for different signals. We have added the following text to the Preprocessing of haplotype data section of the Materials and Methods:

“First, following [55], we used the resizing algorithm 462

from the skimage[58] python package to force a fixed width of 128 for each image 463

(“image resizing”). The algorithm first applies a Gaussian filter to smooth the blocks of 464

the image and avoid aliasing artifacts. Then, it performs a spline interpolation of order 465

1 for downsampling with ‘reflect’ padding applied to the boundaries.” 466

- l.193, l.225: Do you mean S2 Table instead of S1?

This was also an oversight; it has been fixed and we thank the reviewer for their attention to detail!

- l.316-319: Don’t mutation and recombination rate variations equally affect sum stats like H?

The reviewer is certainly right that most summary statistics will have some sensitivity to parameters like the mutation and recombination rates. The text referenced here has been changed, both in response to this question, and also in response to Reviewer #3. The new text clarifies that the main issue with the number of segregating sites is that it is a direct estimate of the mutation rate, and thus particularly sensitive to the mutation rate used for training. The text now reads as follows:

“We find that the image-width standardization preprocessing step in particular has 366

the potential to sway the learning strategy of the CNNs, especially in the context of 367

simulated data with a fixed mutation rate, as is fairly common. We see that 368

standardizing via zero-padding results in a feature that is very salient to the CNNs: the 369

wider blocks of zeros required to pad sweep images relative to neutral images. As a 370

result, the CNNs performs quite well on simulated data, though this performance would 371

likely decline in genuine genome data in which the mutation rates vary. We therefore 372

recommend the deliberate generation of training simulations based on more complex 373

demographic models that allow variable mutation rates if this method of image-width 374

standardization is to be used in preprocessing.” 375

We note also some new text in the Results section in which we discuss the number of segregating sites (which we now call S, to match accepted notation), which is relevant to this discussion and we will excerpt here:

“We note here that this performance is likely inflated relative to 254

what we would expect in real data. This is because our simulated data uses a fixed 255

mutation rate, and S, as a sufficient statistic for the population scaled mutation rate[44] 256

will be extremely sensitive to this parameter by design. In genuine genomes, by 257

contrast, we see a vast range of mutation rates as a function of sequence context and 258

other genome features[2]. Since the CNNs working with zero-padded data appear to be 259

extracting a similar signature, we would also urge caution in interpreting the high 260

accuracies of those models as well. We recommend that a CNN pipeline using 261

zero-padding in preprocessing should be careful to include variable mutation rate 262

parameters in the simulated training data to limit overfitting.” 263

- l.319-321: This could possibly be argued as a strength for ML method, which can use simulations to cover complex scenarios and possibly discover robust features. This is something sum stats cannot do.

We appreciate this point about the strengths of supervised ML in this context, and we have added the following text to the Discussion accordingly:

“This raises the possibility that more complex, tailored models could detect signatures 335

that are as-yet unobserved, given the right architecture and simulation scheme. In 336

particular, we note that the supervised nature of CNNs allows for these models to be 337

trained on complex demographies and evolutionary scenarios to approximate real-world 338

data and draw out nuanced signals in ways that summary statistics may not be able to.” 339

- l.324: Typo? What is “[Otherwise]”?

We thank the reviewer for catching this. It has been removed.

- S1&S2 tables: The presentation of model accuracies is hard to follow, especially considering these results support the key conclusions of this paper. One possibility could be using line-and-point plots (e.g. https://seaborn.pydata.org/tutorial/categorical.html#point-plots) to make the trend stand out. Also, it might help to include the plot as one of the main figures, since these results are central to the narrative.

We appreciate this point, and we are particularly grateful for the suggestion of adding a panel to the main text figures. We have added panel c to Figure 1, which directly compares the performance of Imagene, mini-CNN, and two other architectures which lose performance due to over-simplification of the model. The caption for the new panel is excerpted here:

“c: Accuracy values on a balanced test set simulated from our

single-population demography (error bars represent 95% confidence intervals).

mini-CNN maintains comparable performance to Imagene despite a much simpler

architecture with fewer layers and fewer, smaller kernels. mini-CNN employs a single

2x1 kernel; we find that swapping this out for a 1x2 kernel reduces performance

significantly, as does reducing mini-CNN any further, for example by removing the Relu

activation function. See S1 Fig and S1 Table for architecture details and intermediate

architectures between Imagene and mini-CNN.”

In addition, we have added two supplementary figures, S4and S5 Figs, which are bar plots representing the accuracies presented in what is now S3 Table, across demographic model and selection coefficient, for easier visualization. We note also that these include the error bars (representing 95% confidence intervals) as requested in a previous comment.

- A top-level README file could help the readers navigating through your GitHub repo (accessed 03/21/2023).

We appreciate the feedback. A README file has been added to the github repository. It contains explanations for the different top-level directories and instructions for reproducing the results of the article.

Reviewer #2: The manuscript explores the inner working of CNN for the detection of selective sweeps. The exposition is clear and complete and I find the insights original enough. I have a just some minor suggestions/requests:

We are grateful for this positive feedback, and we have endeavored to address the comments from the reviewer below!

1) There is a big gap in performance between the two selected selection coefficients, with the 0.005 coefficient being very hard to detect for all methods. Did you try to use more haplotypes as input to see if they can increase the detection power of some methods? I think that would be an interesting addition to your work.

We agree that an exploration of the potential of additional haplotypes for increasing power is interesting here. To this end, we have simulated data using our single-population demographic model, generating 1000 haplotypes instead of 128. Previously, with the original data with 128 haplotypes, our preprocessing procedures resized the images to size 128x128. Increasing the image size adds to the total number of parameters within the model and number of convolution operations done in parallel, and hence, requires a larger amount of memory. Due to GPU memory constraints, we were unfortunately unable to resize the newly generated images similarly to a size of 1000x1000. Instead, they were resized to 200x200. Even with the loss of information due to resizing, as expected, we do find that with more data, the model accuracies are improved. The accuracies are presented in a new supplementary table (S4 Table) with the original 128-haplotype results presented alongside for comparison. Importantly, the performance of Garud’s H also improves with the additional data, and the overall relative performances are maintained: Imagene and mini-CNN perform similarly, with neither outperforming the other across the board, while Garud’s H has the highest performance for both selection coefficients. We have added text to reference this observation in Results:

“In Table 1, we compare the accuracy of the CNN approaches and Garud’s H1 for the 169

single-population demographic model. For two selection coefficients representing low 170

and moderate selection strength, s=0.005 and s=0.01, Garud’s H1 actually outperforms 171

both Imagene and mini-CNN, a pattern that is maintained even when these models are 172

trained on images with a larger number of haplotypes (see S4 Table).” 173

We have also added the following details to the Methods section:

“We also performed an experiment simulating 1000 chromosomes from a single 436

population following the same demographic model to validate the patterns found in this 437

manuscript. Original images were resized to 200x200 due to GPU memory constraints. 438

Accuracy results for models trained on this dataset can be found in S4 Table; in short, 439

the results mirror our results for the original 128-chromosome dataset.” 440

2) Please spruce up a bit the github repository: provide a readme with a couple of notes on how to run the code, add a requirement file or at least a list of needed modules, and maybe remove or complete the empty script (reproduce.sh) at the top level.

We appreciate the feedback. A README file has been added to the github repository. It contains explanations for the different top-level directories and instructions for reproducing the results of the article. In addition, the repository now has a requirements.txt file which details the used modules.

3) If possible try to get the figures and tables closer to the text where they are mentioned, it would help the readers!

We understand the frustration here. The PLoS guidelines request the figures to be uploaded separately upon resubmission; we hope that having these separately available will make the next round easier! In addition, we have tried to make sure the figure placeholders/captions are closer to the relevant places in the text.

Reviewer #3: This paper by Cecil and Sugden is concerned with the accuracy and interpretability of deep neural networks (DNN) for population genetic inference. Here, the authors examine the performance of a fairly similar convolutional neural network (CNN), a type of DNN that has been applied to various population genetic tasks in recent years. The authors find that, on their test problem, the CNN performs no better than a simple summary statistic, and also point out ways in which the input representation affects performance.

I enjoyed this paper's emphasis on the importance of interpretability, and their discussion of using CNNs as more than just black boxes, an area in which our field is clearly lagging behind. However, I have some concerns about the generality of the results. Indeed, a close examination of the authors' claims and how they relate to what has previously been shown in the literature strongly suggests that these results do not generalize across CNNs. Nor should we expect these results to generalize across problems, as the authors are looking only at very recent positive selection. I do appreciate the authors' concern about artifacts in deep learning, as this has been well documented in other contexts, and is an issue we should be concerned with in population genetics. But as I argue below, this manuscript contains no analyses that touch upon this subject, instead containing a very misleading argument about the number of segregating sites within a genomic window.

I think that there are important arguments to be made about the tradeoff between interpretability and statistical power, and also it is almost certainly the case that for some tasks more interpretable methods will perform as well as black box approaches (i.e. those problems for which we have a large number of statistics available, and there is a statistic or combination thereof that is likely to perform well in the parameter range of interest, as is certainly the case in sweep detection). Unfortunately, this paper does not make any such arguments very effectively, and offers little in the way of convincing data.

We are grateful for the reviewer’s feedback, and appreciative that they see the need that we do for interpretation in the field of CNNs applied to genome inference. We have taken the author’s critiques to heart, and have made a number of revisions to the paper in response, including many revisions that clarify our intentions and restrict any apparent generalizations. The largest change we have made in response to this review is the way in which we deal with the results concerning the number of segregating sites in a window. The reviewer is entirely correct that this is a valid signature, and we now present it as such. We think the paper is much stronger for it, and that the narrative is also clearer, since this result is now in parallel with the row-sorting/Garud’s H result in that both are ways in which preprocessing decisions present the CNN with the signature we want it to find. Please see below for our responses point by point. Note that excerpts have line numbers for easy reference, and new text is presented in red.

Because I feel that this paper has a number of fundamental flaws, I have focused my review only on major concerns, which I detail below:

1) First, the author's description of the history of sweep detection is misleading, painting a picture that the only methods were summary stats based on the SFS, prior to the advent of the EHH statistic, inflating the importance of the latter. There is no mention of LD-based tests (e.g. https://pubmed.ncbi.nlm.nih.gov/9215920/), and especially Hudson's haplotype test (https://pubmed.ncbi.nlm.nih.gov/8013910/), based on shared haplotypes. There were SFS-based and non-SFS-based statistics devised both before and after the advent of EHH, with may of the latter having nothing to do with EHH. I think this is important here because we want to accurately convey that there have been a vast array of approaches for sweep detection that have attacked the problem from many different angles, and this is relevant to my point above about sweep detecting being a problem that already has a large number of interpretable features that one could use without relying on a deep neural network.

We appreciate the reviewer pointing us towards some areas of the field that were left out of our introduction. We agree that the vast array of approaches is important, and we have added text to the introduction to provide a more complete picture, including the specific point that our current suite of tools is large, even without the addition of deep neural networks. The opening paragraph of the Introduction now reads as follows:

“Over the last few decades, methods for detecting positive selection from present-day 2

genome data have become more sophisticated and more powerful, yielding a large suite 3

of tools designed for this particular evolutionary scenario. Early summary statistics 4

were designed to detect the signature of strong selection, or a “selective sweep” based on 5

its effect on the site frequency spectrum, relying on a signature of over-abundance of 6

low- and high-frequency derived alleles [54, 11]. These were followed by tests 7

capitalizing on the signature of elevated linkage disequilibrium across the site of a sweep, 8

as calculated by allele frequency correlations[27, 60]. Later, the availability of phased 9

genetic data paved the way for the broader use of statistics based on haplotype 10

frequency[42, 24], and then for summary statistics designed to detect extended 11

haplotype homozygosity (EHH)[46, 57, 15, 47, 12]. The 2010s saw the development of 12

composite classification models designed to increase the power to detect sweeps by 13

combining multiple summary statistics in various supervised classification frameworks, 14

training these classifiers on simulated genomic data[48, 20, 53, 51, 34, 41].” 15

The reviewer’s point that the sweep detection problem is one that has a large number of interpretable features already is one we absolutely agree with, and indeed provides the motivation for this study, which aims to challenge the impulse to apply deep neural networks for problems in which other interpretable methods perform as well. We have tried to clarify our intentions, with the following changes to a later paragraph in the introduction:

“The recently proposed state-of-the-art deep learning approaches for detecting 34

selective sweeps are convolutional neural networks (CNNs) [14, 55, 9, 6]. These 35

approaches vary widely in their choice of architecture, including the number of layers, 36

the number and size of convolution kernels, and methods of regularization (see S1 File). 37

While all methods report high accuracy on simulated testing data (typically generated 38

in the same way as the training data), what is as yet unexplored is how these models 39

work, what preprocessing decisions they might be sensitive to, and what insights they 40

might be able to provide to our current theory. In the field of sweep detection, given the 41

wide range of tools already available, and the well-studied theory developed over 42

decades, one possibility is that the complexity of CNNs is unneccessary for improving 43

performance. On the other hand, if they do improve upon our current suite of tools, 44

then by understanding what additional information is deemed valuable by these 45

complex models, it could be possible to improve the design of easy-to-use summary 46

statistics beyond their current capabilities.” 47

2) The authors may well have a CNN that performs no better than Garud's H1 on their task of detecting sweeps, but one cannot generalize based on this observation in the way that the authors are clearly intending. Indeed, there is a clear demonstration that some CNNs perform VASTLY better than H12. For example, the Flagel et al. CNN performs at least as well as a S/HIC, a method based on a vector of summary statistics and which in turn shows a huge increase in accuracy relative to H12 (e.g. Figures 2 and 3 from https://journals.plos.org/plosgenetics/article?id=10.1371/journal.pgen.1005928). (I know that H12 is not the same as H1, but they are so similar and the difference in power between the two is pretty negligible, so that does not matter here.)

We have spent some thought on the discordance between our results here and those referenced by the reviewer in Schrider and Kern 2016, which develops an extra-trees classifier (S/HIC) and Flagel *et al.* 2018, which develops a CNN that performs on par with S/HIC, and is trained on the same coalescent simulations. Schrider and Kern do show that S/HIC outperforms Garud’s H12 in their ROC curves. We acknowledge that it can be difficult to compare studies because of the many differences in demographic models, and the fact that S/HIC is designed to classify into multiple categories: hard sweep, soft sweep, linked, and neutral. That said, the difference in window sizes stands out as a potential explanation for the discrepancy we see. Window statistics like Garud’s H will be sensitive to the window size as a function of the footprint of the sweep. In Garud *et al.* they target window sizes of 10kb to match the theoretical footprint of sweeps in their Drosophila population of interest. Harris *et al.* (2018) estimate footprints on the order of 40kb for non-African human populations; using the same theoretical formula for our single-population demographic model, we expect similar footprints on the order of 50kb. Our simulations span 80kb, while the simulations used for S/HIC are much larger, with summary statistics calculated on 200kb windows, despite also using human demographic models. Since Garud’s H works on haplotype frequencies, by including a large number of sites beyond the sweep footprint, the power of Garud’s H might be expected to decrease dramatically. We have added the following text to Results to make a point about this window sensitivity:

“We note here that in evaluating the performance of Garud’s H1, along with other 174

window-based summary statistics, it is important to consider the window size as it 175

relates to the expected sweep footprint. Following Harris et al.[22], and using our 176

single-population demographic model parameters, we estimate a sweep footprint on the 177

order of 50kb for s = 0.01. Our simulations, which span 80kb, should therefore 178

represent a realm that allows for good, if not optimal, summary statistic performance. 179

With a much larger window size, their performance might be expected to decline, while 180

we may expect the CNNs to maintain their performance and learn to ignore columns 181

outside of the sweep footprint.” 182

We note also that in the interest of full transparency, we provide all of our code for the CNNs and Garud’s H, and all performance analysis on our github page, which now contains instructions for replicating all experiments.

3) Related to point 2 above: the fact that at least some CNNs are much more powerful than Garud's H1, means that the conclusion the authors have reached that "in the process of row-sorting, the CNN is being set up to mimic the behavior of Garud’s H1" clearly cannot be true in general, even for CNNs that sort rows by similarity. It is true that not all CNNs have sorted in the same manner is Imagene, but again, so that may matter somewhat, but the point is that the authors are overgeneralizing and drawing conclusions that are clearly already contradicted in the literature.

We refer to our answer to point 2) above with respect to our findings that contradict other findings in the literature.

We absolutely do not intend to overgeneralize, and we appreciate the opportunity to clarify this. We have added the following paragraph to our Introduction to make our intentions unambiguous upfront:

“We note, finally, that our analyses here are restricted only to the case of recent hard 60

selective sweeps. CNNs have been shown to be effective across a wide range of 61

applications even within evolutionary biology, including for detecting introgression[18], 62

soft sweeps[28], and balancing selection[25]. We hope that this work can serve as a case 63

study for how these other CNNs might be explored and mined for insights. We do not 64

intend to generalize our particular results to those scenarios.” 65

We have made a few similar clarifications in the Discussion to make it clear that our analysis is focused specifically on recent hard sweeps.

4) One's chosen summary stat(s) may not be appropriate given the problem at hand. For example, in some parameterizations (e.g. older sweeps), Garud's H1 will have very little power. If you know exactly which statistic or combination thereof will be most powerful in the relevant region of the parameter space, then this is not much of a problem, as you can choose your statistic(s) appropriately. But this may not always be the case in practice…

We agree that different summary statistics will be appropriate for different regions of parameter space, so as noted above, we have clarified throughout that we are looking specifically at recent hard sweeps. In addition, we have added text to the discussion about this issue, including the benefit of supervised ML that it can be trained specifically on different areas of parameter space:

“On the other hand, all of the models we implemented lose significant 325

performance when selection coefficients become weaker, especially in the context of 326

more complex demographic backgrounds. A similar pattern might be expected with 327

older sweeps, or selection over a much longer timescale, which might necessitate 328

different summary statistics, and would require different training simulations for the 329

CNNs.” 330

[...]

“This raises the possibility that more complex, tailored models could detect signatures 335

that are as-yet unobserved, given the right architecture and simulation scheme. In 336

particular, we note that the supervised nature of CNNs allows for these models to be 337

trained on complex demographies and evolutionary scenarios to approximate real-world 338

data and draw out nuanced signals in ways that summary statistics may not be able to.” 339

5) And what if we don't even have an adequate set of summary statistics for the task at hand? Consider the introgression problem from Flagel et al, where a CNN performs far better than a method using a large vector of summary statistics. The authors pay some lip-service to this possibility, but in a fairly dismissive tone. This is unfortunate, because I think this is probably the real benefit of CNNs: even if for your problem/parameterization we have not already discovered an adequate set of statistics, we have a way forward. Perhaps the authors are trying to argue that we should instead spend more time developing such statistics so we can engineer better feature vectors, and while I would be sympathetic to this argument, I do not find that the analyses in this paper would offer convincing support for it.

We did not intend to be dismissive here, and we regret that it read that way. We agree that CNNs are powerful, both for providing a way forward in the absence of effective summary statistics, and for providing insights that might lead to better summary statistics. We have changed some of the language in the Discussion to emphasize this point:

”To this point, CNNs have been shown to be effective in other population genetics 340

applications including identifying recombination hotspots[3] and adaptive 341

introgression[18], and distinguishing between soft sweeps and balancing selection[25], 342

none of which we explore here. We also note that there are more complex architectures 343

that exist in the literature, including a branched architecture that takes in positional 344

data in addition to the haplotype image[14], potentially allowing for the model to learn 345

more nuanced signatures. This additional model complexity makes interpretation more 346

difficult, so we have not attempted it here. In all of these cases, the additional 347

complexity of the CNNs may well be beneficial, especially in areas with less robust 348

suites of summary statistics than the hard sweep case. Ideally, by examining these 349

CNNs, insight could be gained that might result in more easily-applied tools in order to 350

make them more broadly available; we hope that this study provides some inspiration 351

for such investigations, which can dovetail with cutting-edge interpretive and 352

explanatory tools as they emerge.” 353

6) Finally, I am somewhat taken aback by the notion that encoding the number of segregating sites in a physical region is somehow artifactual. The density of polymorphism is of course informative about natural selection, and the authors seem to be implying that the researchers who chose to design CNNs that very clearly represent this information were somehow accidentally "artificially inflating" performance, a notion that strains credulity. It is quite trivial to point out that this padding approach essentially includes the number of segregating sites as an input feature. Indeed, some network architectures have a separate input channel that in some way encodes the position of segregating sites, and when that is padded the number of segregating sites is even more readily extracted by the network. Do the authors truly believe that in these cases the designers of those networks were unaware that this would be happening. This is obviously not the case, and the number of segregating sites is instead intentionally being given to the network because it is useful for answering the question at hand, as regions affected by positive selection will have relatively few polymorphisms. Of course, there may be some situations where one does not want to use this information, and the authors could make an argument about this if they wish. But, to me, the blanket implication that a network’s encoding of the number of polymorphisms in a window is somehow undesirable or unintended is almost akin to stating that Watterson's estimator contains entirely artifactual information. In short, the authors’ unnecessarily provocative claim about so-called artifactual information is confusing, distracting, and completely unsupported, and I strongly urge the authors to remove it.

We are particularly grateful for this comment from the reviewer. We have considered it thoroughly, and we have incorporated this perspective in place of our discussion of shortcut learning. We see the similarity of CNNs trained on zero-padding to Watterson’s theta (or just S, the number of segregating sites), as a parallel to our results about row-sorting and Garud’s H. We have restructured the relevant section in Results, newly named “Effects of image-width standardization on CNN learning strategies,” to make this more nuanced point. There are too many changes to excerpt here, so we will refer to this new results section in its entirety.

In addition, we have removed our discussion of shortcut learning throughout (see Abstract, Author Summary, Results, Figure 4 caption, and Discussion), as we agree that this is not entirely correct in this context. What we have retained is a discussion of how fixed-mutation-rate simulation schemes may lead to inflated performance, and recommendations for how to address this if researchers wish to use zero-padding as a preprocessing step.

In short, despite stating early on in the Introduction that "the choice of CNN architecture varies widely across these works" and that this in part "makes direct comparison between the approaches tricky", the authors have used a very small number of analyses on a single CNN to make a straw-man argument, which they then overgeneralize from to imply that sets of statistics, or even single statistics, have more power than neural networks (which may be the case for some especially easy parameterizations of especially well-studied problems), even though their results and conclusions clearly do not hold water when examined in the context of previously published results in this area.

We thank the reviewer for giving the manuscript a thoughtful review, and for identifying important places where the text needed clarification. We hope that our changes outlined above have addressed the reviewer’s overall critiques. In particular, we have endeavored to make sure that we are not generalizing our results beyond the sweep context, and to express our intentions more clearly. We have also attempted to provide plausible explanations for the discrepancy between some of our results and some others in the published literature, and we hope that together with our transparency via github, this addresses that specific concern.

**Have the authors made all data and (if applicable) computational code underlying the findings in their manuscript fully available?**
The [PLOS Data policy](https://nam02.safelinks.protection.outlook.com/?url=https%3A%2F%2Fjournals.plos.org%2Fploscompbiol%2Fs%2Fmaterials-and-software-sharing&data=05%7C01%7Csugdenl%40duq.edu%7Cc6639b96541e4928f8b408db3e8b1c8e%7C12c44311cf844e4195c38df690b1eb61%7C0%7C0%7C638172540019517009%7CUnknown%7CTWFpbGZsb3d8eyJWIjoiMC4wLjAwMDAiLCJQIjoiV2luMzIiLCJBTiI6Ik1haWwiLCJXVCI6Mn0%3D%7C3000%7C%7C%7C&sdata=BHkP5bFxKFM5b0231dZl0OfBIl4QR5n4wfLVsGzuEv8%3D&reserved=0) requires authors to make all data and code underlying the findings described in their manuscript fully available without restriction, with rare exception (please refer to the Data Availability Statement in the manuscript PDF file). The data and code should be provided as part of the manuscript or its supporting information, or deposited to a public repository. For example, in addition to summary statistics, the data points behind means, medians and variance measures should be available. If there are restrictions on publicly sharing data or code —e.g. participant privacy or use of data from a third party—those must be specified.

Reviewer #1: Yes

Reviewer #2: Yes

Reviewer #3: None

PLOS authors have the option to publish the peer review history of their article ([what does this mean?](https://nam02.safelinks.protection.outlook.com/?url=https%3A%2F%2Fjournals.plos.org%2Fploscompbiol%2Fs%2Feditorial-and-peer-review-process%23loc-peer-review-history&data=05%7C01%7Csugdenl%40duq.edu%7Cc6639b96541e4928f8b408db3e8b1c8e%7C12c44311cf844e4195c38df690b1eb61%7C0%7C0%7C638172540019517009%7CUnknown%7CTWFpbGZsb3d8eyJWIjoiMC4wLjAwMDAiLCJQIjoiV2luMzIiLCJBTiI6Ik1haWwiLCJXVCI6Mn0%3D%7C3000%7C%7C%7C&sdata=DovJpKISAC1ZcHHTcbHHFmJL06IgoyrsWDhPriMzNrQ%3D&reserved=0)). If published, this will include your full peer review and any attached files.


If you choose “no”, your identity will remain anonymous but your review may still be made public.


**Do you want your identity to be public for this peer review?** For information about this choice, including consent withdrawal, please see our [Privacy Policy](https://nam02.safelinks.protection.outlook.com/?url=https%3A%2F%2Fwww.plos.org%2Fprivacy-policy&data=05%7C01%7Csugdenl%40duq.edu%7Cc6639b96541e4928f8b408db3e8b1c8e%7C12c44311cf844e4195c38df690b1eb61%7C0%7C0%7C638172540019517009%7CUnknown%7CTWFpbGZsb3d8eyJWIjoiMC4wLjAwMDAiLCJQIjoiV2luMzIiLCJBTiI6Ik1haWwiLCJXVCI6Mn0%3D%7C3000%7C%7C%7C&sdata=917N2Er8Iz15bco9XiGhg1hG38mCv%2Bxr1QMRnMKES%2Bk%3D&reserved=0).

Reviewer #1: No

Reviewer #2: No

Reviewer #3: No

Figure Files:

While revising your submission, please upload your figure files to the Preflight Analysis and Conversion Engine (PACE) digital diagnostic tool, [https://pacev2.apexcovantage.com](https://nam02.safelinks.protection.outlook.com/?url=https%3A%2F%2Fpacev2.apexcovantage.com%2F&data=05%7C01%7Csugdenl%40duq.edu%7Cc6639b96541e4928f8b408db3e8b1c8e%7C12c44311cf844e4195c38df690b1eb61%7C0%7C0%7C638172540019517009%7CUnknown%7CTWFpbGZsb3d8eyJWIjoiMC4wLjAwMDAiLCJQIjoiV2luMzIiLCJBTiI6Ik1haWwiLCJXVCI6Mn0%3D%7C3000%7C%7C%7C&sdata=xjc3l7dWGtb30BYvXFSbQ01GJCAR1glLNGEmfCxIZIA%3D&reserved=0). PACE helps ensure that figures meet PLOS requirements. To use PACE, you must first register as a user. Then, login and navigate to the UPLOAD tab, where you will find detailed instructions on how to use the tool. If you encounter any issues or have any questions when using PACE, please email us at figures@plos.org.

Data Requirements:

Please note that, as a condition of publication, PLOS' data policy requires that you make available all data used to draw the conclusions outlined in your manuscript. Data must be deposited in an appropriate repository, included within the body of the manuscript, or uploaded as supporting information. This includes all numerical values that were used to generate graphs, histograms etc.. For an example in PLOS Biology see here: http://www.plosbiology.org/article/info%3Adoi%2F10.1371%2Fjournal.pbio.1001908#s5.

Reproducibility:

To enhance the reproducibility of your results, we recommend that you deposit your laboratory protocols in protocols.io, where a protocol can be assigned its own identifier (DOI) such that it can be cited independently in the future. Additionally, PLOS ONE offers an option to publish peer-reviewed clinical study protocols. Read more information on sharing protocols at [https://plos.org/protocols?utm_medium=editorial-email&utm_source=authorletters&utm_campaign=protocols](https://nam02.safelinks.protection.outlook.com/?url=https%3A%2F%2Fplos.org%2Fprotocols%3Futm_medium%3Deditorial-email%26utm_source%3Dauthorletters%26utm_campaign%3Dprotocols&data=05%7C01%7Csugdenl%40duq.edu%7Cc6639b96541e4928f8b408db3e8b1c8e%7C12c44311cf844e4195c38df690b1eb61%7C0%7C0%7C638172540019517009%7CUnknown%7CTWFpbGZsb3d8eyJWIjoiMC4wLjAwMDAiLCJQIjoiV2luMzIiLCJBTiI6Ik1haWwiLCJXVCI6Mn0%3D%7C3000%7C%7C%7C&sdata=Kvmx7UJ%2BjG6prXJoErFT1ZXm6fgSB3r0KJ%2FAVKFBpFQ%3D&reserved=0)
