# Supplementary material for: On convolutional neural networks for selection inference: Revealing the effect of preprocessing on model learning and the capacity to discover novel patterns
Source: PLoS Comput Biol. 2023 Nov 27;19(11):e1010979. doi: 10.1371/journal.pcbi.1010979 (PMC10703409; doi:10.1371/journal.pcbi.1010979)
Supplement: Attachment — Submitted filename: PCOMPBIOL_revision_review.docx [file pcbi.1010979.s005.docx]

I believe the authors have adequately addressed all reviewers’ comments with updates in the discussion that thoroughly examine the caveats and limitations of this study, as well as by carrying out several additional analyses suggested, which is very much appreciated.

The manuscript is in very good shape and I have only one major suggestion for the authors and editors to consider – the significant revision to the original submission seems to warrant an updated title. In light of the changes to the manuscript in response to comments from reviewer #3 that pointed out some major caveats against interpreting the results pertaining to summary statistics, I am not sure if the emphasis on summary statistics in the title is relevant any longer. This article reads to me like a cautionary tale of how the capacity of deep learning methods are under-explored due to human preprocessing, where summary statistic is only a small part of it. And I think the title should reflect this.

Minor comments:

- Regarding this comment from the authors: “*In particular, to maintain the performance of the full Imagene model, four 3x3 kernels are necessary. To the reviewer’s point above, this indicates that a lack of sorting makes 1D kernels much less effective compared to 2D kernels.*”

I apologize that I misused the word “kernel” in my original comment. Instead of these 3x1, 1x3, 1x2 kernels etc. (which the authors call 1D kernels), I actually meant 1D convolution **layers**. A 1D conv layer would actually have kernels that are **large** enough that they only convolve in a single direction, which are typically of size W x N, where W = width, N = # of haplotypes. For example, 3 x 128 kernels so for simulations in this study with 128 haplotypes. Figure 1 in Flagel et al. (2019) actually has a very good graphical illustration of this if you compare panels B (2D conv filter) & C (1D conv filter).

So my original point was that 1D **layers** could be more robust when haplotypes are unsorted since they act on whole columns, across ALL haplotypes, and are more suited to discover global features, whereas 2D **layers** (which are exclusively used in this study) have smaller **kernels** that extract local features are more prone to the effects of sorting.

While I still think it is an important and interesting hypothesis to be tested, this could be beyond the scope of this paper, and it is my fault of not communicating this precisely. (Although it might be worth acknowledging in the paper that the analyses do not apply to CNN architectures 1D conv **layer** architectures)

- I appreciate the authors following through with the additional experiments that compare different training strategies! If possible, could you offer a bit more detail of your implementation of simulation-on-the-fly (s.o.t.f.) in the methods section? What do you mean by “single epoch” (in the figure caption), in the end what is the total number of simulations when training is done?

The reason I am curious is because I am a bit surprised by the result that s.o.t.f. is underperforming despite in theory it’s supposed to be the best approach. “*[L]imited [only] by computational time and space,* “*[s]imulation-on-the-fly" uses this ability to generate almost unlimited training data to prevent overfitting, as it involves carrying out simulations alongside training, so each data point is only seen once during training. This continuous simulation of data is carried out for many training iterations, until validation loss is sufficiently small*” (Torada et al.). It is usually deployed together with early stopping where you only stop producing additional simulations when the val loss stops decreasing. In theory, it is expected to perform better than using only early stopping with a fixed amount of training data.

- Fig. 1 panel c, figs S4&S5: you might want to truncate the y-axis for better visuals of the differences b/n models.
- Could you put the standard error calculations in the methods section? There is some rendering issue in the reply letter to the reviewer, and can’t seem to see the equation properly.
