# Supplementary material for: On convolutional neural networks for selection inference: Revealing the effect of preprocessing on model learning and the capacity to discover novel patterns
Source: PLoS Comput Biol. 2023 Nov 27;19(11):e1010979. doi: 10.1371/journal.pcbi.1010979 (PMC10703409; doi:10.1371/journal.pcbi.1010979)
Supplement: Attachment — Submitted filename: response_to_reviewers.docx [file pcbi.1010979.s006.docx]

Dear Mr. Cecil,

Thank you very much for submitting your manuscript "On convolutional neural networks for selection inference: revealing the lurking role of preprocessing, and the surprising effectiveness of summary statistics" for consideration at PLOS Computational Biology.

As with all papers reviewed by the journal, your manuscript was reviewed by members of the editorial board and by several independent reviewers. In light of the reviews (below this email), we would like to invite the resubmission of a significantly-revised version that takes into account the reviewers' comments.

We cannot make any decision about publication until we have seen the revised manuscript and your response to the reviewers' comments. Your revised manuscript is also likely to be sent to reviewers for further evaluation.

When you are ready to resubmit, please upload the following:

[1] A letter containing a detailed list of your responses to the review comments and a description of the changes you have made in the manuscript. Please note while forming your response, if your article is accepted, you may have the opportunity to make the peer review history publicly available. The record will include editor decision letters (with reviews) and your responses to reviewer comments. If eligible, we will contact you to opt in or out.

[2] Two versions of the revised manuscript: one with either highlights or tracked changes denoting where the text has been changed; the other a clean version (uploaded as the manuscript file).

Important additional instructions are given below your reviewer comments.

Please prepare and submit your revised manuscript within 60 days. If you anticipate any delay, please let us know the expected resubmission date by replying to this email. Please note that revised manuscripts received after the 60-day due date may require evaluation and peer review similar to newly submitted manuscripts.

Thank you again for your submission. We hope that our editorial process has been constructive so far, and we welcome your feedback at any time. Please don't hesitate to contact us if you have any questions or comments.

Sincerely,

Piero Fariselli
Academic Editor
PLOS Computational Biology

James O'Dwyer
Section Editor
PLOS Computational Biology

***********************

Dear Editor,

We are grateful for your help in guiding this manuscript through the review process. We have done our best to address the reviewers’ comments and are happy to present our revised manuscript. In response to the reviewers, we have made changes throughout, including to the title, to better articulate our purpose in this manuscript, which is not to criticize deep learning in this field, but to investigate how we might, as population geneticists, learn from these models in order to make their insights more broadly available, and how preprocessing decisions may affect this goal. We have also expanded our discussion of CNNs from the literature to place our case study more soundly in context, including discussion of the “simulation-on-the-fly” training, and the architectures and simulation strategy of the sweep model in Flagel *et al.* Lastly, we have added further comments on the great potential of deep learning methods in more complex evolutionary scenarios.

Reviewer's Responses to Questions

**Comments to the Authors:
Please note here if the review is uploaded as an attachment.**

Reviewer #1: Review is uploaded as an attachment.

I believe the authors have adequately addressed all reviewers’ comments with updates in the discussion that thoroughly examine the caveats and limitations of this study, as well as by carrying out several additional analyses suggested, which is very much appreciated.

The manuscript is in very good shape and I have only one major suggestion for the authors and editors to consider – the significant revision to the original submission seems to warrant an updated title. In light of the changes to the manuscript in response to comments from reviewer #3 that pointed out some major caveats against interpreting the results pertaining to summary statistics, I am not sure if the emphasis on summary statistics in the title is relevant any longer. This article reads to me like a cautionary tale of how the capacity of deep learning methods are under-explored due to human preprocessing, where summary statistic is only a small part of it. And I think the title should reflect this.

We are grateful for the reviewer’s comments throughout this review process. We agree with the need to revisit the title to better emphasize the themes of the updated manuscript. Our new title is as follows:

“On convolutional neural networks for selection inference: revealing the effect of preprocessing on model learning and the capacity to discover novel patterns.”

Minor comments:

- Regarding this comment from the authors: “*In particular, to maintain the performance of the full Imagene model, four 3x3 kernels are necessary. To the reviewer’s point above, this indicates that a lack of sorting makes 1D kernels much less effective compared to 2D kernels.*”

I apologize that I misused the word “kernel” in my original comment. Instead of these 3x1, 1x3, 1x2 kernels etc. (which the authors call 1D kernels), I actually meant 1D convolution **layers**. A 1D conv layer would actually have kernels that are **large** enough that they only convolve in a single direction, which are typically of size W x N, where W = width, N = # of haplotypes. For example, 3 x 128 kernels so for simulations in this study with 128 haplotypes. Figure 1 in Flagel et al. (2019) actually has a very good graphical illustration of this if you compare panels B (2D conv filter) & C (1D conv filter).

So my original point was that 1D **layers** could be more robust when haplotypes are unsorted since they act on whole columns, across ALL haplotypes, and are more suited to discover global features, whereas 2D **layers** (which are exclusively used in this study) have smaller **kernels** that extract local features are more prone to the effects of sorting.

While I still think it is an important and interesting hypothesis to be tested, this could be beyond the scope of this paper, and it is my fault of not communicating this precisely. (Although it might be worth acknowledging in the paper that the analyses do not apply to CNN architectures 1D conv **layer** architectures)

We appreciated the opportunity to dig into the Flagel et al. architecture more deeply because we were also confused by the layer/kernel issue, and had misinterpreted the kernels as 2x1 (similar to mini-CNN) rather than nx2. We have corrected this misconception in our discussion, and following the reviewer’s suggestion, we acknowledge the use of this architecture as a path that we do not explore in this manuscript in the excerpt below. We think that the question of whether such an architecture would be more or less robust to sorting is an open and interesting one; on the one hand, the sorting may matter more since the model is learning row-specific weights for each kernel, but it is possible that by combining the output of multiple such kernels, more general patterns can be learned.

“We also note that there are more complex architectures	343

that exist in the literature, including a branched architecture that takes in positional	344

data in addition to the haplotype image and employs kernels that span all haplotypes at	345

once[14], potentially allowing for the model to learn more nuanced signatures.”	346

- I appreciate the authors following through with the additional experiments that compare different training strategies! If possible, could you offer a bit more detail of your implementation of simulation-on-the-fly (s.o.t.f.) in the methods section? What do you mean by “single epoch” (in the figure caption), in the end what is the total number of simulations when training is done?

The reason I am curious is because I am a bit surprised by the result that s.o.t.f. is underperforming despite in theory it’s supposed to be the best approach. “*[L]imited [only] by computational time and space,* “*[s]imulation-on-the-fly" uses this ability to generate almost unlimited training data to prevent overfitting, as it involves carrying out simulations alongside training, so each data point is only seen once during training. This continuous simulation of data is carried out for many training iterations, until validation loss is sufficiently small*” (Torada et al.)

We agree that the general term ‘simulation on the fly’ may be misleading for this approach and have changed our description of the strategy to better emphasize that we were implementing the training strategy of the Imagene paper where they introduce the concept. The original code that we used can be found here: <https://github.com/mfumagalli/ImaGene/blob/master/Reproduce/train_binary.py>.

Briefly, they first simulate 100,000 images, then do a single pass through 80,000 of them for training. This may be considered as ‘simulation on the fly’ where training is manually set to stop after 80,000 simulations have been used, with the benefit being that the model cannot overfit the training data by doing multiple passes. We refer to this single pass as one epoch to match the general machine learning literature, in which an “epoch” is typically defined as a single pass through the training dataset during training. We agree with the reviewer that what we implemented was not a true simulation and early stopping strategy in which we simulated until a specific metric stopped decreasing. The results of our sample complexity analysis, however, indicate that if we were to implement a true ‘simulation on the fly’ approach (where we simulated data until the validation loss stopped decreasing) which should, in theory, have the best performance, we would expect it to have similar performance to our currently trained models. This can be seen by the diminishing returns in performance as the sample size increases in Supplementary Figure S16. Based on these analyses, the fact that the Torada strategy occasionally underperforms the other two training strategies may come down to the fact that by only doing one pass, the model has had fewer opportunities to update its parameters, resulting in incomplete convergence relative to the other training strategies on the same amount of data.

We have updated the main text Methods (Implementation of Deep Learning Models) to be more precise about the simulation schemes we implemented:

“To ensure that this training scheme is appropriate, we have also	500

implemented two other strategies from the literature: early stopping, and the strategy	501

used in Torada *et al.* for ImaGene[55], which they refer to as simulation on-the-fly, and	502

evaluated model accuracy (S17 Fig, S18 Fig). We see that our strategy performs on par	503

with early stopping, and on average performs better than the ImaGene strategy	503

We have also updated the supplementary figure captions S17 and S18 to provide more detail about our implementation along with the caveats for interpretation of the relative performance:

"Each sub-plot corresponds to a different demographic model, the x-axis denotes the model type, the y-axis corresponds to the accuracy of the model on the balanced, held-out set, and the color of the bar corresponds to the training strategy. We refer to an epoch as a single training pass through the training dataset. The ``Best of 10'' training strategy trains the model 10 times for 2 epochs each, then selects the best performing model from the 10 trainings using the validation set. The ``Early Stopping'' strategy trains the model for 100 epochs, and utilizes early stopping to stop training whenever the validation accuracy fails to increase after 2 epochs of training. The ``Simulation on the Fly'' strategy follows the strategy used in Torada *et al.*[1], which trains the model for a single epoch, meaning that each training simulation is used only once. This approach may be viewed as simulating samples on the fly where the total number of simulated samples was decided upon prior to training. The early stopping and simulation on the fly trainings were restarted if the model failed to improve in accuracy after the first epoch. Across all the demographic models and selection coefficients, the range of accuracy differences between the early stopping and best of 10 strategies were (-0.0599, 0.0445), (-0.0725, -0.0019), and (-0.097, 0.0137) for Imagene, Mini-CNN, and Deepset, respectively. The same range of accuracy differences between the simulation on the fly and best of 10 strategies were (-0.1595, 0.0100), (-0.1956, 0.0155), and (-0.0490, -0.0078). We note that this implementation of ``Simulation on the fly'' may be at a disadvantage in this comparison because the additional epochs of the other training strategies result in more updates to the model during training; while it is possible to continue simulating novel data for simulation on the fly, our sample complexity analyses (see S16 Fig) indicate that we would likely see diminishing returns and would not expect this to result in much higher accuracy than we see with the other training strategies.”

- Fig. 1 panel c, figs S4&S5: you might want to truncate the y-axis for better visuals of the differences b/n models.

We agree that this would make the differences more stark, but we are concerned that this would be more misleading than helpful. Most data visualization best practices warn against truncating the y-axis when comparing magnitudes as in this case (for example, refer to Duke University Libraries best practices here: <https://guides.library.duke.edu/datavis/topten>).

- Could you put the standard error calculations in the methods section? There is some rendering issue in the reply letter to the reviewer, and can’t seem to see the equation properly.

We apologize for the formatting issue (and apologize in advance if it happens again here). We have added the relevant equation to the Methods section (Implementation of Deep Learning Models), where it should render correctly in the pdf. We have not included the excerpt here, because it will not appear as it should in the word document.

Reviewer #3: In their revision, Cecil and Sugden have added several helpful analysis to their paper, "On convolutional neural networks for selection inference: revealing the lurking role of preprocessing, and the surprising effectiveness of summary statistics." More importantly, the authors state in their response that they have toned down their claims, which I appreciate. However, upon carefully examining the actual changes made in the manuscript, I found that there were key points in the manuscript that are still misleading:

We are grateful for the reviewers feedback; we think our manuscript is much stronger for the more nuanced discussion of the number of segregating sites and how different summary statistics align with different patterns recognized by the CNN. We have done our best to address the remaining concerns. In particular, we have changed and expanded text in places to better articulate our intentions with this article. We do not intend this as a criticism of neural networks in the realm of population genetics. On the contrary, since they have been demonstrated to be so effective, our primary goal is to learn from them if possible. The analyses that follow in the paper arise fundamentally from this goal; in order to learn from these models, we want to understand how they make decisions, and how design decisions in preprocessing affect model learning. Many of the changes that are described below were made in an effort to clarify this.

1) Saying that they cannot conclude that their findings would generalize to other problems (or more challenging formulations of the sweep detection problem) is not sufficient, as it leaves the door open to the possibility that their findings might generalize, when it is in fact quite clear that they would not.

Although the authors pay lip service to the fact that there are more statistics for detecting sweeps than for some other problems (e.g. detecting introgressed loci), a more complete discussion of what is going on in these other problems is vitally important as this would better characterize the real motivation for using deep learning in population genetics: not only is there not a single statistic that can recapitulate the performance of a DNN for detecting introgression, even a combination of such statistics does not can get anywhere close. Until such statistics have been designed, deep learning will be a more powerful alternative. In other words, the motivation is that one need not wait for the ideal statistic or set thereof to be derived, but if and when those statistics are discovered, more interpretable methods with similar performance may be possible (and preferable).

We are in agreement with the reviewer that neural networks play an important and exciting role in population genetics, especially in cases where summary statistics are less well-developed than they are for selective sweeps. What we aim to suggest here is that perhaps these models can do *even* *more* for us, in terms of pointing us in new directions that might be fruitful for developing summary statistics that can be put to use more widely by those without the computational resources or training to implement complicated models. We have tried to make this clearer up front in the Introduction as follows:

More recently, advances in machine learning have presented an exciting prospect:	16

these models might be able to learn patterns of natural selection directly from raw data,	17

thereby extracting more information than is available through current handcrafted	18

summary statistics. To this end, multiple deep learning architectures have recently been	19

proposed that can detect signatures of natural selection from sequence data with high	20

accuracy[48, 30]. In addition, deep learning has been applied to other population	21

genetics problems such as identifying recombination hotspots[3] and adaptive	22

introgression[17], and distinguishing between soft sweeps and balancing selection[24].	23

Especially in these areas, where the well of theoretically-driven summary statistics is	24

less deep than for selective sweeps, models that can learn new patterns and carry out	25

these tasks without reliance on statistics provide an exciting way forward. Beyond	26

achieving high performance at these tasks, we further propose that these models can	27

help point us in useful directions: through advances in deep learning interpretation	28

methods [34, 42, 44], we have the potential to conceive of new easy-to-implement	29

summary statistics based on learned features.	30

2) I was surprised to see that the authors were still insinuating that neural networks that contain information about the density of polymorphism are in fact using unintended/artifactual information, when this is clearly not the case. Researchers making the design choices to include padding/positional information know that their input contains the density of segregating sites. The authors can state that they wish to inform those who may be new to neural network design about this, which would be useful, but manuscript still seems to be implying that previous studies were unintentionally using this information, and this needs to be corrected. Indeed, the title's mention of the "lurking role" of preprocessing contributes to this unnecessarily accusatory tone and I find it to be unjustified and inappropriate.

We certainly do not intend to be accusatory at any point, so we have updated the manuscript to make sure this never appears to be the case. We have removed all language that might have implied that researchers are using information that is artifactual. In particular, we have changed the title of our manuscript so that it no longer refers to the “lurking” role of preprocessing, and we have removed all language in the Introduction about the potential for overfitting based on features that do not generalize well, or “over-reliance” on the number of segregating sites. We have also added language to emphasize that researchers can make preprocessing decisions deliberately in order to introduce useful information. See in particular changes to the Introduction, Results, and Discussion, including the following excerpts from the Introduction, which used to include text about overfitting and over-reliance on the number of segregating sites respectively:

While deep learning approaches have become state-of-the-art for a variety of tasks 31

such as image processing [32, 31, 22, 29], natural language processing [58, 7, 1], game 32

playing [39, 51], and protein folding [49, 25], these models are often highly complex and 33

not well understood. A few consequences of this are worth consideration. First, there 34

are infinitely many possible model architectures, and identifying the optimal 35

architecture for a given problem can be difficult. Second, once an architecture is chosen, 36

it can be difficult to explain the resulting model’s predictions. Third, deliberate 37

preprocessing decisions that determine how data is presented to the model can affect 38

the decision rules learned by the model. On one hand these decisions often improve 39

model performance by allowing the model to access known signals within the data. On 40

the other hand, these decisions can limit the model to focusing only on a previously 41

known signal, thereby constraining the model from learning something new. 42

…Using these tools, we 62

show empirically that typical preprocessing pipelines induce the CNN models for sweep 63

detection to closely mimic handcrafted summary statistics; depending on how the data 64

is presented to the models, their decision rules are particularly similar to Garud’s H1, 65

which typically performs at least as well at this classification task, or they pay closest 66

attention to the number of segregating sites in a window, mimicking one of the earliest 67

and simplest measures of genetic diversity, Watterson’s theta. 68

Please see also our related changes in response to the next point about Flagel *et al.*

On a related note, It is helpful that the authors point out the importance of training data with variable mutation rates, but make no effort to mention previous studies that have done this (e.g. for the sweep-detection problem in Flagel et al., theta varied across an order of magnitude).

Thank you for the note; we had indeed overlooked this. We have included a mention of the Flagel et al. article in the results section where we discuss this:

We recommend that a CNN pipeline using zero-padding in 270

preprocessing should be careful to include variable mutation rate parameters in the 271

simulated training data, as is done in Flagel et al.[14] to limit overfitting. 272

In addition, we have also included this fact in our Discussion on the effects of pre-processing at the end of the article:

We find that the image-width standardization preprocessing step in particular has 371

the potential to sway the learning strategy of the CNN. We see that standardizing via 372

zero-padding results in a feature that is very salient to the CNNs: the wider blocks of 373

zeros required to pad sweep images relative to neutral images. This is particularly 374

extreme in our simulations, which do not have a variable mutation rate in contrast to 375

the application in Flagel et al.[14].

3) Yes, when developing a new method, design decisions matter, and good designers will make those intentionally and carefully. The authors' premise seems to be that researchers using deep learning are putting no thought into their designs, and simply outsourcing all of their decision making to the training process. I strongly disagree with this view. For example, deep learning gives designers far greater flexibility in designing input representations. In the abstract, the authors say, "We conclude that human decisions still wield significant influence on these methods," which is of course obvious and well known. It would be more accurate to say that your study examines some of the ways that some of these decisions can influence performance.

We have made fundamental changes to our title, abstract, and author summary, along with changes to the manuscript, to better articulate what we intend to communicate with this article. This includes removal of the abstract sentence noted by the reviewer. We agree that researchers are often very thoughtful in their implementations, and our goal here is to examine how those decisions manifest in terms of the decision rules learned by the CNN. In addition, we refer to the changes we have made in response to the comments above relating to how we discuss model design decisions.

Abstract:

For evolutionary processes that are less well

understood than selective sweeps, we hope this provides an initial framework for using

CNNs in ways that go beyond simply achieving high classification performance. Instead,

we propose that CNNs might be useful as tools for learning novel patterns that can

translate to easy-to-implement summary statistics available to a wider community of

researchers.

Author Summary:

We present here a case study, examining these issues in the context of a central problem in

population genetics: detecting patterns of selection from genome data. Through this

application, we show how human decision-making can encourage the model to see what

we want it to see in various ways. By understanding how these models work, and how

they respond to the particular way in which data is presented, we have a chance of

creating new frameworks that are capable of discovering novel patterns.

4) I also want to point out that the authors' arguments about window size and H12 are unconvincing, given that Garud et al. (2015) examined H12 in 400-SNP windows.

We are unclear where the disagreement arises here. In the Garud 2015 paper, they do target 400-SNP windows, “corresponding roughly to 10 kb in the DGRP data.” This 10kb span arises precisely from the formula for calculating the footprint of a hard sweep given a set of demographic parameters that they reference in their paper ( *s*/[log(*Nes*)*ρ*] ), which we use to justify a ~50kb footprint for a hard sweep in human populations. The smaller footprint for *Drosophila melanogaster* relative to human populations seems to be a result of their much larger effective population size (>10^6 according to Garud *et al.* compared to <10^4 for human populations).

**Have the authors made all data and (if applicable) computational code underlying the findings in their manuscript fully available?**
The [PLOS Data policy](https://nam02.safelinks.protection.outlook.com/?url=https%3A%2F%2Fjournals.plos.org%2Fploscompbiol%2Fs%2Fmaterials-and-software-sharing&data=05%7C01%7Csugdenl%40duq.edu%7Ce3e79c4d411d4e8accdd08db9c55530d%7C12c44311cf844e4195c38df690b1eb61%7C0%7C0%7C638275662696908808%7CUnknown%7CTWFpbGZsb3d8eyJWIjoiMC4wLjAwMDAiLCJQIjoiV2luMzIiLCJBTiI6Ik1haWwiLCJXVCI6Mn0%3D%7C3000%7C%7C%7C&sdata=eqrRs%2Bq1AiyeEdUa1yymTXCVllTtTwuovFQzE9GJKGw%3D&reserved=0) requires authors to make all data and code underlying the findings described in their manuscript fully available without restriction, with rare exception (please refer to the Data Availability Statement in the manuscript PDF file). The data and code should be provided as part of the manuscript or its supporting information, or deposited to a public repository. For example, in addition to summary statistics, the data points behind means, medians and variance measures should be available. If there are restrictions on publicly sharing data or code —e.g. participant privacy or use of data from a third party—those must be specified.

Reviewer #1: Yes

Reviewer #3: None

PLOS authors have the option to publish the peer review history of their article ([what does this mean?](https://nam02.safelinks.protection.outlook.com/?url=https%3A%2F%2Fjournals.plos.org%2Fploscompbiol%2Fs%2Feditorial-and-peer-review-process%23loc-peer-review-history&data=05%7C01%7Csugdenl%40duq.edu%7Ce3e79c4d411d4e8accdd08db9c55530d%7C12c44311cf844e4195c38df690b1eb61%7C0%7C0%7C638275662696908808%7CUnknown%7CTWFpbGZsb3d8eyJWIjoiMC4wLjAwMDAiLCJQIjoiV2luMzIiLCJBTiI6Ik1haWwiLCJXVCI6Mn0%3D%7C3000%7C%7C%7C&sdata=6urXCOkXHn2CccerzYDPd33L0n9%2BkJvGRc%2BNoaCT0yA%3D&reserved=0)). If published, this will include your full peer review and any attached files.


If you choose “no”, your identity will remain anonymous but your review may still be made public.


**Do you want your identity to be public for this peer review?** For information about this choice, including consent withdrawal, please see our [Privacy Policy](https://nam02.safelinks.protection.outlook.com/?url=https%3A%2F%2Fwww.plos.org%2Fprivacy-policy&data=05%7C01%7Csugdenl%40duq.edu%7Ce3e79c4d411d4e8accdd08db9c55530d%7C12c44311cf844e4195c38df690b1eb61%7C0%7C0%7C638275662696908808%7CUnknown%7CTWFpbGZsb3d8eyJWIjoiMC4wLjAwMDAiLCJQIjoiV2luMzIiLCJBTiI6Ik1haWwiLCJXVCI6Mn0%3D%7C3000%7C%7C%7C&sdata=DgNIztBvJKNA%2F4JpKTdLy1LGg7w88%2F%2B4hgC8PCMgc8w%3D&reserved=0).

Reviewer #1: No

Reviewer #3: No

Figure Files:

While revising your submission, please upload your figure files to the Preflight Analysis and Conversion Engine (PACE) digital diagnostic tool, [https://pacev2.apexcovantage.com](https://nam02.safelinks.protection.outlook.com/?url=https%3A%2F%2Fpacev2.apexcovantage.com%2F&data=05%7C01%7Csugdenl%40duq.edu%7Ce3e79c4d411d4e8accdd08db9c55530d%7C12c44311cf844e4195c38df690b1eb61%7C0%7C0%7C638275662696908808%7CUnknown%7CTWFpbGZsb3d8eyJWIjoiMC4wLjAwMDAiLCJQIjoiV2luMzIiLCJBTiI6Ik1haWwiLCJXVCI6Mn0%3D%7C3000%7C%7C%7C&sdata=ItKnYH%2FR2RSiZLhBu%2BJSqnjl4PWd2gQxBM5NScPOHsI%3D&reserved=0). PACE helps ensure that figures meet PLOS requirements. To use PACE, you must first register as a user. Then, login and navigate to the UPLOAD tab, where you will find detailed instructions on how to use the tool. If you encounter any issues or have any questions when using PACE, please email us at figures@plos.org.

Data Requirements:

Please note that, as a condition of publication, PLOS' data policy requires that you make available all data used to draw the conclusions outlined in your manuscript. Data must be deposited in an appropriate repository, included within the body of the manuscript, or uploaded as supporting information. This includes all numerical values that were used to generate graphs, histograms etc.. For an example in PLOS Biology see here: http://www.plosbiology.org/article/info%3Adoi%2F10.1371%2Fjournal.pbio.1001908#s5.

Reproducibility:

To enhance the reproducibility of your results, we recommend that you deposit your laboratory protocols in protocols.io, where a protocol can be assigned its own identifier (DOI) such that it can be cited independently in the future. Additionally, PLOS ONE offers an option to publish peer-reviewed clinical study protocols. Read more information on sharing protocols at [https://plos.org/protocols?utm_medium=editorial-email&utm_source=authorletters&utm_campaign=protocols](https://nam02.safelinks.protection.outlook.com/?url=https%3A%2F%2Fplos.org%2Fprotocols%3Futm_medium%3Deditorial-email%26utm_source%3Dauthorletters%26utm_campaign%3Dprotocols&data=05%7C01%7Csugdenl%40duq.edu%7Ce3e79c4d411d4e8accdd08db9c55530d%7C12c44311cf844e4195c38df690b1eb61%7C0%7C0%7C638275662696908808%7CUnknown%7CTWFpbGZsb3d8eyJWIjoiMC4wLjAwMDAiLCJQIjoiV2luMzIiLCJBTiI6Ik1haWwiLCJXVCI6Mn0%3D%7C3000%7C%7C%7C&sdata=q2%2BmLk92FVvQPYX4c3eDvSdlNFQkApbXJmIjrGllfMA%3D&reserved=0)

*In compliance with data protection regulations, you may request that we remove your personal registration details at any time.* [*(Remove my information/details)*](https://nam02.safelinks.protection.outlook.com/?url=https%3A%2F%2Fwww.editorialmanager.com%2Fpcompbiol%2Flogin.asp%3Fa%3Dr&data=05%7C01%7Csugdenl%40duq.edu%7Ce3e79c4d411d4e8accdd08db9c55530d%7C12c44311cf844e4195c38df690b1eb61%7C0%7C0%7C638275662696908808%7CUnknown%7CTWFpbGZsb3d8eyJWIjoiMC4wLjAwMDAiLCJQIjoiV2luMzIiLCJBTiI6Ik1haWwiLCJXVCI6Mn0%3D%7C3000%7C%7C%7C&sdata=Ue%2BoSYHDfYB2a2ANqm0XDCHzupCKKsgSSgGcmqYoCGY%3D&reserved=0)*. Please contact the publication office if you have any questions.*
